# Photo-amygdalin: light-dependent control over hydrogen cyanide release and cytotoxicity†

**DOI:** 10.1039/d5sc01248a

**Published:** 2025-05-09

**Authors:** Albert Marten Schulte, Ainoa Guinart, Georgios Alachouzos, Wiktor Szymanski, Ben L. Feringa

**Affiliations:** a Centre for Systems Chemistry, Stratingh Institute for Chemistry, Faculty for Science and Engineering, University of Groningen Nijenborgh 4 9747 AG Groningen The Netherlands b.l.feringa@rug.nl w.c.szymanski@rug.nl; b Department of Medicinal Chemistry, Photopharmacology and Imaging, Groningen Research Institute of Pharmacy, University of Groningen Antonius Deusinglaan 1 9713 AV Groningen The Netherlands; c Department of Radiology, Medical Imaging Center, University Medical Center Groningen, University of Groningen Hanzeplein 1 9713 GZ Groningen The Netherlands

## Abstract

Amygdalin is a natural glycosidic compound found in bitter almonds and apricot seeds. After enzymatic hydrolysis, amygdalin forms a cyanohydrin which spontaneously decays to release toxic hydrogen cyanide in a process called cyanogenesis. Due to this capacity to release cyanide, it has a long and controversial history of use as an anticancer therapeutic in alternative medicine. However, the inability to control amygdalin's cyanogenesis hinders its efficient medical use and renders it potentially dangerous. In this study we describe the design and development of ‘photo-amygdalin’, a compound whose cyanogenesis can be triggered by visible light. We synthesize photo-amygdalin inspired by the molecular structure of amygdalin, illustrate its capability to form hydrogen cyanide upon irradiation through a myoglobin test, and showcase its ability to significantly reduce the viability of a human cell line upon irradiation, proving it to be 100-fold more potent than potassium cyanide.

## Abstract 2

A photoresponsive analogue of amygdalin enables light-dependent control over hydrogen cyanide release, myoglobin cyanide binding and cytotoxicity.

## Graphical abstract

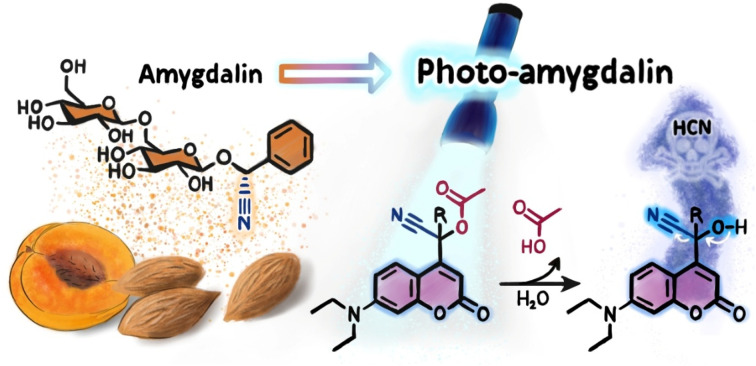

## Introduction

Cancer is a major global health threat, most notably in the developed world.^1^ In 2020, around 10 million people died of this disease, and new cases are projected to rise worldwide by 47% yearly by 2040.^2^ Given this large disease burden, extensive efforts are devoted to research towards the eradication of tumor tissue. Besides surgical intervention, the main strategies used for tumor-treatment are radiation in radiotherapy and the use of cytotoxic agents in chemotherapy. Chemotherapy relies both on synthetic as well as natural compounds, with widely used Taxol being an example of the latter.^3^ A major problem with current chemotherapy remains the selectivity of targeting tumor tissue over healthy tissue. Since most chemotherapeutical agents lack this selectivity, they cause severe side effects that often significantly reducing a patients' quality of life.^4^

**Fig. 1:**
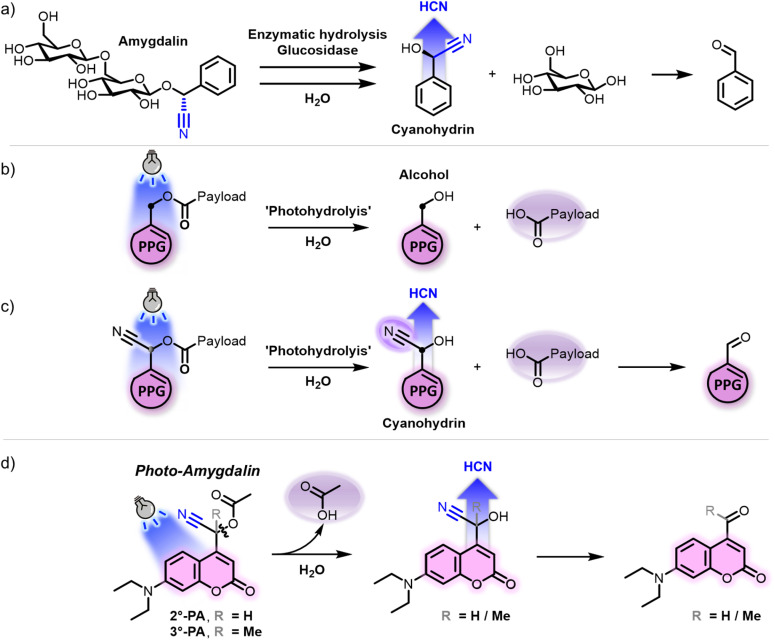
Theoretical basis and design of photo-amygdalin. (a) Schematic representation of the enzymatic hydrolysis of amygdalin, resulting in the formation of the cyanohydrin mandelonitrile and subsequent cyanogenesis. (b) Schematic representation of the ‘photohydrolysis’ process occurring after irradiation of a heterolytic photocleavable protecting group (PPG), resulting in formation of the PPG-alcohol and liberation of the payload. (c) Installation of a cyano-substituent at the PPG alpha-carbon would, under irradiation, result in payload release and formation of a cyanogenic cyanohydrin. (d) Proposed design of a modified diethylamino-coumarin PPG that upon irradiation would release cyanohydrin.

Cyanogenic glycosides are a class of natural compounds that have a controversial history of use in chemotherapy.^5^ These plant-derived molecules characteristically feature a carbohydrate and cyanohydrin moiety, connected *via* a glycosidic bond.^6^ Perhaps the most widely known cyanogenic glycoside is amygdalin, a compound found in high concentrations in almonds, apricot seeds and peach seeds.^7^ Whereas amygdalin itself is a stable compound, enzymatic hydrolysis of its β-glycosidic linkage results in the formation of a labile cyanohydrin (Fig. 1a).^8^ Then, in a process called cyanogenesis, the formed cyanohydrin dissociates into benzaldehyde and hydrogen cyanide. Cyanide is infamous for its toxicity stemming from its ability to inhibit metalloenzymes, in particular cytochrome c oxidase (*i.e.* complex IV), which is an enzyme in the mitochondrial electron transport chain.^9,10^

Amygdalin, together with its semi-synthetic analogue laetrile, has a long history of use in alternative medicine for the treatment of cancer.^11^ Proponents of its efficacy claim that amygdalin specifically targets tumor cells over regular cells, a hypothesis that thus far remains unproven.^5^ The popularity of amygdalin led to an investigation into its efficacy and a clinical trial performed in 1982 dubbed amygdalin ‘a toxic drug that is not effective as a cancer treatment’.^12^ On the other hand, amygdalin's efficacy to limit the proliferation of cancer cells or even induce their apoptosis *in vitro* has been demonstrated.^7,11,13,14^

Given that the crucial step in the activation of amygdalin's cytotoxicity is enzymatic hydrolysis leading to cyanogenesis, amygdalin's degree of cytotoxicity depends largely on the (over)expression of beta-glucosidase, the enzyme catalyzing this process. A study in which beta-glucosidase was specifically targeted to tumor cells significantly increased the cytotoxicity of amygdalin, illustrating that the natural activity of the enzyme –and therefore the natural antitumor effect of amygdalin– is limited.^15^ Furthermore, since the distribution of this enzyme is non-specific to tumor cells, cyanogenesis remains untargeted as well. Therefore, using amygdalin alone to selectively target cancer cells will be ineffective and cyanide poisoning resulting from untargeted cyanogenesis in healthy tissue will remain a considerable risk.

In this work, we aimed to design and evaluate a molecular system that would allow a highly precise control over the release of cytotoxic cyanide. Drug activation and targeting can be achieved in a variety of ways, such as pH-dependent activation^16^ or conjugation with antibodies.^17^ However, we reasoned that a system depending on light-activation would allow a particularly high degree of control. For example, in photopharmacology, the use of light-controlled drugs allows for their activation in space and time with high accuracy simply by directing the irradiation source,^18–22^ a degree of control that's unattainable using pH- or antibody-dependent strategies.

The crucial step in cyanogenesis is the hydrolysis of the glycosidic bond, which liberates the unstable cyanohydrin moiety. Fundamental to the design of our photochemical tool, we recognized that hydrolysis of a bond could also be achieved photochemically using heterolytic photocleavable protecting groups (PPGs). Upon irradiation in water, a PPG of this type undergoes a form of photo-hydrolysis of the bond between the payload and the PPG alpha-carbon, to form the PPG-alcohol and liberate the payload (Fig. 1b).^23–25^ Building on this reactivity pattern, we envisioned that, when a cyano-group is installed at the PPG alpha-carbon, the resulting PPG photoproduct would not be an alcohol but a cyanohydrin (Fig. 1c). Analogously to the formed cyanohydrin in the case of amygdalin, this photoproduct should be unstable and undergo cyanogenesis, a process that therefore could hypothetically be activated by light, non-invasively and with high spatial–temporal precision. Additionally, whereas in the case of amygdalin the hydrolysis product besides the cyanohydrin is glucose (Fig. 1a), the use of a PPG would mean that the second ‘photohydrolysis’ product would be its payload (Fig. 1c). Conveniently, this payload could be chosen freely and could be any small molecule of interest. For example, in the field of photopharmacology, PPGs have been widely used to release bioactive (chemotherapeutic) payloads upon irradiation, allowing for their light-controlled activation.^18–21,26,27^ Overall, the conceptual cyano-PPG displayed in Fig. 1c would liberate two distinct payloads upon irradiation.

While in principle any member of the class of heterolytic PPGs could be used to test the hypothesis outlined in Fig. 1c, we decided to use diethylamino-coumarin PPGs. Coumarin PPGs are one of the oldest members of the class of heterolytic PPGs and are synthetically easily accessible. Furthermore, they undergo visible light activation (violet-blue light, *λ* = 400 nm) and feature higher photolysis quantum yields (QYs) than other members of the class of heterolytic PPGs that respond to green- or red-light.^28–30^ We therefore designed coumarin based photo-amygdalin compounds featuring a cyano-substituent at the PPG alpha-carbon and bearing a model acetic acid (AcOH) payload (Fig. 1d). Furthermore, we compared the performance of both the classical secondary coumarin PPG (Fig. 1d, 2°-PA) and its tertiary analogue (Fig. 1d, 3°-PA), inspired by our recent discovery of the increased uncaging quantum yields featured by tertiary PPGs in which the carbocation formed after uncaging is stabilized through hyperconjugation.^25^

## Results and discussion

### Synthesis

**Fig. 2:**
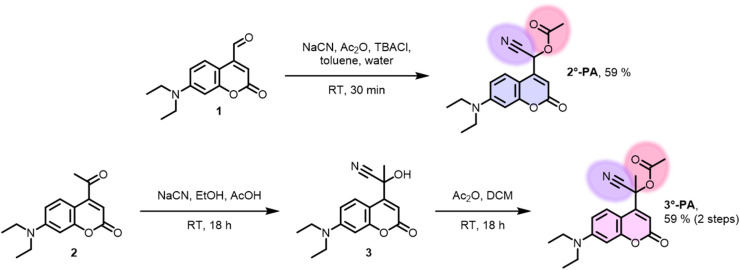
Synthesis of the photo-amygdalins 2°-PA and 3°-PA.

Often, the last step in the synthesis of PPG-payload conjugates is the reaction between the desired payload and the PPG-alcohol.^31^ However, in the case of 2°-PA and 3°-PA, the PPG bearing a hydroxyl functionality would not be a simple benzylic alcohol, but a labile cyanohydrin. Therefore, we initially sought a way to avoid the necessity for isolation of the cyanohydrin intermediate. Inspired by a literature procedure,^32^ a biphasic system was used containing NaCN, acetic anhydride and a phase-transfer catalyst. Starting from coumarin-aldehyde 1, this yielded the acetylated cyanohydrin 2°-PA conveniently in one step (Fig. 2). Formation of tertiary analogue 3°-PA using this procedure proved unsuccessful, arguably due to the reduced electrophilicity of ketone 2 as compared to the aldehyde. Therefore, we decided to test a two-step procedure, first forming the cyanohydrin intermediate 3 using NaCN in a mixture of ethanol and acetic acid. While the formed intermediate 3 was indeed unstable and slowly decomposed back to the ketone, it was sufficiently stable to endure a workup. Subsequent acetylation using acetic anhydride in DCM yielded the desired compound 3°-PA. Both 2°-PA and 3°-PA were sufficiently stable to be isolated and characterized successfully.

### Photochemical evaluation of 2°-PA and 3°-PA

**Fig. 3:**
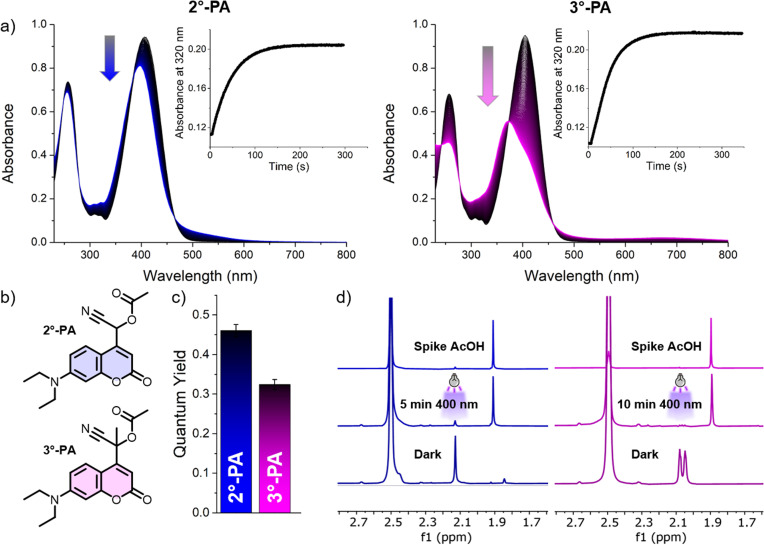
Photochemical evaluation of 2°-PA and 3°-PA. (a) UV-vis absorption spectra of 2°-PA (left) and 3°-PA (right, both at 55 μM concentration in 97 : 3 (v/v) water/MeCN, 25 °C) upon irradiation (*λ*_max_ = 390 nm, total irradiation time 5 min, photon flux 7.05 × 10^−6^ mmol s^−1^), the insert shows the change in absorption at 320 nm upon irradiation. (b) Structures of 2°-PA and 3°-PA (c) Quantum yields of PPG conversion of 2°-PA and 3°-PA (*n* = 3, determined from (a)). (d) Partial ^1^H-NMR spectra of 2°-PA and 3°-PA (2 mM, DMSO-d_6_/D_2_O 1 : 1) in the dark (bottom) and after irradiation with violet light (*λ*_max_ = 400 nm, 5 min for 2°-PA, 10 min for 3°-PA, middle spectrum). The release of AcOH was confirmed through a spike with AcOH (top spectrum). For 3°-PA, two singlets are observed in the dark, stemming from the acetate and methyl protons. Complete spectra can be found in the ESI.†

With the secondary and tertiary model photo-amygdalins in hand, we set out to study their photochemical properties. Irradiation of solutions of both compounds in water with violet light (*λ* = 390 nm) led to remarkably fast changes in their absorption spectra, and stable spectra were obtained after only seconds of irradiation. To study the process in more detail, the irradiation intensity was lowered (photon flux lowered ∼5-fold from 33.5 × 10^−6^ to 7.05 × 10^−6^ mmol s^−1^) and absorption spectra were recorded upon irradiation (Fig. 3a). Interestingly, 3°-PA showed a pronounced change in the shape of the spectrum upon irradiation, hinting at the formation of compounds structurally significantly different from 3°-PA and therefore indicating a complex photochemical mechanism. HPLC-MS analysis of irradiated samples of 2°-PA and 3°-PA revealed that for both compounds no single major PPG-derived photoproduct was formed, but a variety of species (ESI Section 2.4†). Whereas the *m*/*z* signal corresponding to the expected ketone photoproduct could be observed in the irradiated sample of 3°-PA, in the irradiated samples of 2°-PA neither the aldehyde nor its hydrate were observed (see ESI Section 2.4†).

The QY of PPG conversion could be determined by UV-vis spectroscopy. To our surprise, these quantum yields were found to be remarkably high, around 46% for 2°-PA and 32% for 3°-PA (Fig. 3c). Given that a cyano-substituent is an electron withdrawing group – as indicated by its strongly positive Hammet *σ*_p_-constant of 0.66 (ref. 33) – we were initially puzzled by these results, since the installation of an electron withdrawing group at the PPG alpha-carbon should reduce stability of the intermediate PPG alpha-cation formed in the photolysis process and therefore reduce the QY.^24,25^ A possible explanation for the remarkably high QY could be cation stabilization through delocalization of the π-electrons of the cyano group. Cyano-substituents have previously been found to stabilize alpha-cations in some cases, and this π-donor effect is higher for unstable cations.^34^ To further test this hypothesis, we performed DFT-calculations to investigate the heterolysis of acetate in the first singlet excited state (S_1_), the state in which the uncaging of coumarin-PPGs is known to occur.^24^ These calculations revealed that the introduction of the cyano-substituent indeed resulted in a significant reduction in the computed energy barriers of acetate heterolysis in S_1_, displaying barrier heights of 5.9 and 8.3 kcal mol^−1^ for 2°-PA and 3°-PA, respectively (see ESI Section 5†). In contrast, analogous coumarin molecules solely lacking the cyano substituent show energy barriers of 12.0 and 9.6 kcal mol^−1^ for the analogues of 2°-PA and 3°-PA, respectively.^25^ Furthermore, while our DFT-calculations were unable to detect a CIP intermediate in S_1_, in T_1_ highly stabilized CIP intermediates were found for both compounds (ESI, Fig. S36†), further hinting on the stabilizing nature of the cyano substituent.

Subsequently, we set out to study whether irradiation of the compounds indeed led to liberation of both payloads: acetic acid and cyanide. ^1^H-NMR analysis in D_2_O/DMSO-*d*_6_ 1 : 1 revealed that upon violet light irradiation (*λ*_max_ = 400 nm), acetic acid was released from both PPGs (Fig. 3d). Detection of the release of cyanide proved to be more challenging. ^1^H-NMR analysis did not allow for its detection since the use of deuterated solvents would lead to the formation of DCN instead of HCN. Furthermore, since we reasoned water to be crucial in the cyanogenesis process through formation of the cyanohydrin intermediate, the requirement for a high water content of the mixture did not allow us to reach a sufficiently high concentration of the PPGs for the detection of the ^13^C-signal of cyanide.

### Cyanide release studied using the myoglobin test

To confirm the release of cyanide, we decided to use UV-vis spectroscopy, a technique that would enable the detection of low concentrations and accordingly allow for high water contents of the mixture. Seeking a method for visualization of cyanide formation, we realized that some metal-complexes reveal the binding of cyanide through changes in their absorption spectrum. One of these complexes is myoglobin, an analogue of hemoglobin responsible for the storage of oxygen in muscle tissue.^35,36^ The Soret-band of myoglobin is sensitive to the presence of different ligands, and its *λ*_max_ value changes depending on which ligand (*e.g.* O_2_, CO or CN) is bound.^37^

**Fig. 4:**
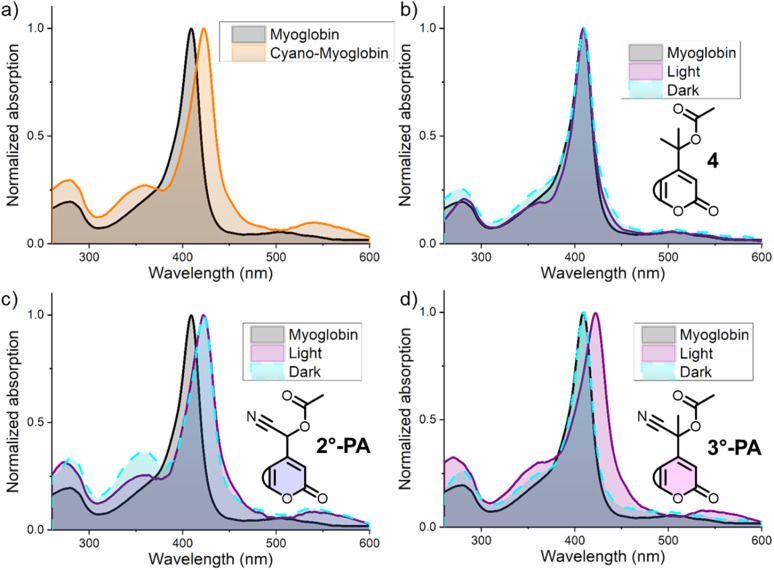
Results of the myoglobin cyanide test. (a) Normalized UV-vis absorption spectra of myoglobin (1.5 μM) in PBS-buffer (pH 7.3) at 25 °C in the absence and presence of cyanide (40 μM). (b–d) UV-vis absorption spectra of myoglobin (1.5 μM) in PBS-buffer (pH 7.3) at 25 °C in the presence of compound 4/2°-PA/3°-PA (50 μM), that was either irradiated (‘light’, *λ*_max_ = 390 nm, 1 min for 2°-PA and 3°-PA, 2 min for 4) or kept in the dark for the same period of time (‘dark’). Also shown is the absorption spectrum of myoglobin as reference. For (b–d), the absorption spectra of compounds 4, 2°-PA or 3°-PA after dark incubation or irradiation was subtracted before the addition of myoglobin. All displayed spectra were recorded after incubation at 25 °C for 10 min.

The addition of NaCN (40 μM) to a solution of myoglobin (1.5 μM) in PBS-buffer, followed by a 10 min incubation period, led to a clear (∼15 nm) bathochromic shift in the absorption spectrum (Fig. 4a), resulting in an absorption peak at 423 nm, corresponding to the absorption spectrum of a myoglobin-cyanide complex reported in literature.^37^ With this positive control confirming cyanide-binding through UV-vis spectroscopy, we set out to study the effect of addition and irradiation of PPGs 2°-PA and 3°-PA on the absorption spectrum of myoglobin. Inconveniently, the absorption spectra of these coumarin compounds also show *λ*_max_ values around 400 nm and therefore would show significant overlap with that of myoglobin, complicating the detection of cyanide-binding. Therefore, we decided to use aqueous solutions of 2°-PA and 3°-PA that were either irradiated or kept in the dark as blank measurements, and subsequently add myoglobin. Initially, these experiments were performed with tertiary coumarin 4,^25^ a control PPG also bearing an acetate payload but lacking the cyano-substituent. The presence of this compound had no effect on the absorption spectrum of myoglobin and both the samples that were kept in the dark or irradiated showed the same *λ*_max_ value as myoglobin itself (Fig. 4b).

Unfortunately, the use of 2°-PA led to the formation of cyano-myoglobin both in dark and irradiated samples, indicating that 2°-PA was not stable for the incubation period (Fig. 4c). This was later confirmed by UV-vis and UPLC-MS studies under prolonged incubation in aqueous solution, which showed a hydrolytic instability of 2°-PA (ESI, Section 3†). In the same experiments, 3°-PA showed high hydrolytic stability, showing just minimal hydrolysis after 48 h at RT. To our delight, irradiation of the 3°-PA samples led to the formation of cyano-myoglobin, whereas in samples that were kept in the dark the natural peak of myoglobin was retained (Fig. 4d). These results demonstrate that for 3°-PA, the desired irradiation-dependent cyanogenesis was achieved and, importantly, cyanogenesis did not occur in the dark in the incubation period.

While this test allows for the detection of cyanide and shows proof of principle, it does not allow for its quantification. Studying whether complete consumption of 3°-PA under irradiation would result in the equimolar formation of cyanide would require a more sensitive cyanide detection method. The release of cyanide is likely partly due to formation of the intermediate labile cyanohydrin, supported by the ketone observed after irradiation of 3°-PA (ESI Section 2.4†). However, at the moment we cannot confirm if other photochemical pathways also contribute to cyanogenesis. Given the formation of a wide range of other photoproducts besides the expected ketone resulting from the irradiation of 3°-PA (ESI Section 2.4†), it is likely that alternative photochemical routes are also contributing to cyanide formation.

In summary, both 2°-PA and 3°-PA release their payloads, HCN and AcOH, upon irradiation with remarkably high quantum yield. Whereas 2°-PA showed hydrolytic instability releasing cyanide in the dark, 3°-PA showed improved hydrolytic stability and no cyanogenesis in the dark. Based on these results, we decided to use 3°-PA featuring the additional methyl group at the alpha-carbon in our further studies.

**Fig. 5:**
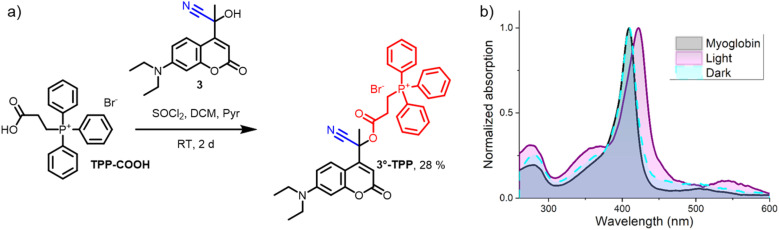
Synthesis and myoglobin cyanide test of 3°-TPP. (a) Synthesis of cyanogenic PPG 3°-TPP and (b) the result of its myoglobin test (1.5 μM myoglobin in PBS-buffer (pH 7.3) at 25 °C in the presence of 3°-TPP (50 μM) either irradiated (‘light’, *λ*_max_ = 390 nm, 1 min) or kept in the dark for 1 min (‘dark’). The absorption spectrum of 3°-TPP after dark incubation or irradiation was subtracted before the addition of myoglobin. All displayed spectra recorded after incubation at 25 °C for 10 min).

Given that the cytotoxic effect of cyanide is a result of a mitochondrial enzyme inhibition, we reasoned that when 3°-PA is accumulated in mitochondria, upon irradiation a higher concentration of cyanide would be released at its site of action, increasing the cytotoxic effect. Mitochondria can be specifically targeted by certain molecular moieties, for example triphenylphosphonium (TPP) compounds are known to accumulate in these organelles.^38,39^ TPP-functionalization of lonidamine, a drug targeting tumor cell glycolysis, resulted in a significant increase in its potency.^40^ Inspired by this observation, we installed the TPP functionality on the tertiary cyano-coumarin 3 through a reaction with TPP-COOH (Fig. 5a). In a one pot procedure, initially the acyl chloride of TPP-COOH was formed followed by the addition of cyanohydrin 3, yielding the cyanogenic triphenylphosphonium PPG 3°-TPP. The myoglobin test confirmed that 3°-TPP shared the favorable properties of 3°-PA and released cyanide solely upon irradiation (Fig. 5b).

### Cell viability assay

**Fig. 6:**
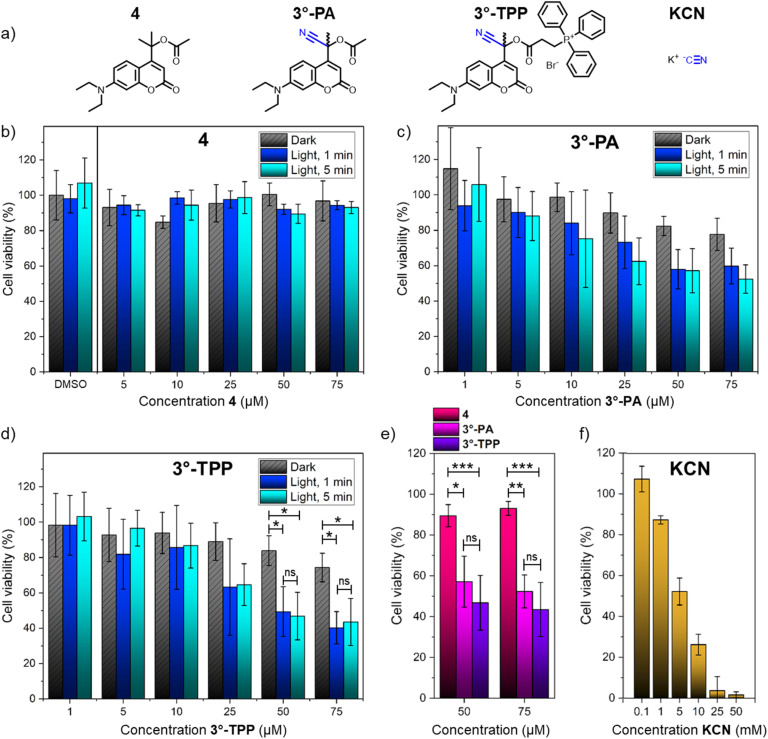
Results of the cell viability assay. (a) Structures of the tested compounds 4, 3°-PA, 3°-TPP and KCN. (b) Results of the cell viability assay for compound 4. (c) Results of the cell viability assay for 3°-PA. (d) Results of the cell viability assay for 3°-TPP. (e) Results of the cell viability assay of 4, 3°-PA and 3°-TPP after 5 min of irradiation. (f) Results of the cell viability assay for KCN. ANOVA tests were performed, and stars indicate *p*-values: * < 0.05, ** < 0.01 and *** < 0.001 or ns > 0.05. Reported are the averages and standard deviations of three independent experiments in which different cell batches were used.

We set out to study the cytotoxicity of the synthesized compounds 3°-PA and 3°-TPP towards human embryonic kidney cells (HEK239). In this assay, the concentrations of both 3°-PA and 3°-TPP, as well as the irradiation times, were varied. Cells were incubated with the photo-amygdalins for 24 h, and subsequently either irradiated (*λ* = 400 nm) for one or five minutes or kept in the dark. Afterwards, cells were incubated at 37 °C for 24 h, and viability was evaluated using a 3-(4,5-dimethylthiazol-2-yl)-2,5-diphenyl-2*H*-tetrazolium bromide (MTT) assay. Furthermore, in these experiments, control compound 4 was included, featuring a tertiary coumarin PPG core bearing an acetic acid payload (Fig. 6a). Just as for 3°-PA, upon irradiation of compound 4 acetic acid is released, but since this compound does not contain a cyanide substituent at the alpha-carbon, no cyanogenesis takes place, as was also confirmed by the myoglobin test (Fig. 6b). Therefore, compound 4 provides the control to establish the effect of irradiation dependent release of acetic acid itself.

In a 24 h incubation period, 3°-PA showed a decent stability in phosphate-buffer at the temperature used in the assay (37 °C), according to UV-vis and UPLC-MS measurements (ESI Section 3†). Unfortunately, 3°-TPP did not display a stable absorption spectrum at this temperature, and UPLC-MS studies also indicated a reduced stability of 3°-TPP in these conditions. Nevertheless, we were interested to see how both compounds would behave in the cell viability assay.

Initially, we established that irradiation of the cells with violet light (*λ* = 400 nm) for 5 min itself had no effect on cell viability (Fig. 6b, DMSO). Furthermore, control compound 4 also had no effect on cell viability at concentrations up to 75 μM, both in the dark and upon irradiation (Fig. 6b). Excitingly, for cyanogenic PPGs 3°-PA and 3°-TPP, the release of cyanide at μM concentrations did reduce the mean cell viability (Fig. 6c and d). Also, irradiation of both cyanogenic PPGs resulted in a significant reduction in mean cell viability as compared to irradiation of compound 4 (Fig. 6e). At a 50 μM concentration and an irradiation time of merely 1 min, 3°-TPP reduced the mean cell viability down to 50% (Fig. 6d).

The statistical significance of the data was evaluated through ANOVA tests. For both compounds, no statistical difference in cytotoxicity between one and five minutes of irradiation was observed (all *p*-values > 0.4, see ESI Section 4.3†), suggesting that the majority of PPGs undergoes photolysis within the first minute of irradiation. These results are in agreement with the high QY that was determined earlier for 3°-PA (Fig. 3c), highlighting the efficiency of these compounds. However, while irradiation of 3°-PA at all tested concentrations did lower the mean cell viability (6c), for this compound the difference between dark and irradiated samples was not found to be statistically significant. Comparing the cytotoxicity of 3°-PA upon irradiation with that of control compound 4 did reveal a statistically significant difference (6e). Gratifyingly, for the mitochondria-targeted 3°-TPP a statistically significant difference in cell viability was found between irradiated and dark samples at 50 and 75 μM (6d). Furthermore, comparing the cytotoxicity of 3°-TPP upon irradiation to that of compound 4 revealed highly significant differences (6e). Although upon irradiation 3°-TPP reduced cell viability to a larger extent than 3°-PA did, this difference was found to be not significant (6e).

While irradiation of both 3°-PA and 3°-TPP resulted in significant cytotoxicity as compared to control compound 4, only for 3°-TPP the effect of irradiation itself was found to be unequivocally established. This indicates that also in the dark, a certain amount of cyanide is released from the cyanogenic compounds. Surprisingly, cytotoxicity in the dark is comparable for both compounds (Fig. 6c and d, grey bars), indicating a comparable stability of 3°-PA and 3°-TPP. Therefore, the reduced stability of 3°-TPP as compared to 3°-PA that was determined in water (ESI Section 3.2†) is not reflected in the results of the cell viability assay. While these results at first glance seem confusing, they could stem from a difference in stability of the compounds once they are taken up by the cell, as compared to their stability in PBS-buffer. While the localization of the coumarin-PPGs inside the cell so far remains unknown, if these lipophilic compounds were to be taken up in the cellular- or mitochondrial-membrane, they would likely feature an increased stability as compared to that in an aqueous medium. After uptake, cellular-stability should be the determining factor rather than hydrolytic stability, and the observed similar dark cytotoxicity could indicate that 3°-PA and 3°-TPP show a similar cellular-stability. Furthermore, we hypothesized that outside of the cell, the instability of 3°-TPP might be less relevant since cyanide formation in the medium would likely have a lower cytotoxic effect than cyanide formation inside the cell after PPG irradiation. Studies in a 2 : 1 MeCN/PBS-buffer mixture showed excellent stability of 3°-PA and even 2°-PA, with both compounds showing no hydrolysis after 48 h, illustrating a potentially higher stability in more hydrophobic environments such as cell membranes (ESI Section 3.2†). In the same study, 3°-TPP also showed high stability, while it degraded rapidly in 99 : 1 water/DMSO, indicating that the higher amount of acetonitrile reduces its hydrolysis rate significantly.

Given these hypotheses, we ultimately set out to study the cytotoxic effects of potassium cyanide (KCN) itself. Using the same procedure for the cell viability assay, the cells were incubated with KCN. Excitingly, as compared to the cyanogenic PPGs 3°-PA and 3°-TPP, a ∼100-fold higher concentration of KCN was required to cause similar cytotoxicity (Fig. 6f). This indicates that cyanide delivery inside the cell constitutes the main bottleneck for its cytotoxicity, a bottleneck that then can be effectively overcome through the use of photo-amygdalins acting as “Trojan horses”, *i.e.*, masked, photoactivatable sources of cyanide.

## Conclusion and outlook

In this study, we present the design, photochemical evaluation and cell toxicity studies of photo-amygdalins. These coumarin-based compounds were designed to feature secondary or tertiary centers at the cyanide-bearing alpha-carbon (2°-PA and 3°-PA). Upon irradiation of these compounds, the payload acetic acid was released, identifying them as a new variant of PPGs. Both PPGs showed surprisingly high QYs of PPG conversion of 46 and 32% for 2°-PA and 3°-PA, respectively. A myoglobin test was developed to evaluate the irradiation-dependent release of HCN. This test revealed that 2°-PA suffered from poor hydrolytic stability and it released cyanide in the dark. In contrast, the more stable 3°-PA displayed the desired property of releasing cyanide solely upon irradiation. Based on the molecular design of 3°-PA, 3°-TPP was developed featuring a lipophilic quaternary phosphonium moiety known to accumulate in mitochondria, the intracellular target of cyanide. A cell viability assay was performed to evaluate the irradiation dependent cytotoxicity of the cyanogenic PPGs. While after only a minute of irradiation of both 3°-PA and 3°-TPP cell viability was reduced, due to dark cytotoxicity only for 3°-TPP the effect of irradiation was found to be statistically significant. 3°-TPP was also slightly more cytotoxic upon irradiation than 3°-PA, an effect that could be due to the mitochondria targeting triphenylphosphonium moiety of 3°-TPP. Interestingly, cyanogenic PPG 3°-TPP was ∼100-fold more cytotoxic than KCN itself, hypothetically stemming from superior cell-penetrating properties of 3°-TPP. Overall, the cell viability assay illustrates that cytotoxicity towards HEK239 cells can be controlled through the photo-release of cyanide when employing 3°-TPP. Furthermore, irradiation of control compound 4 confirmed that the observed cytotoxicity of 3°-TPP is indeed caused by cyanogenesis resulting from its irradiation.

In the future, photo-amygdalin compounds may be used as a chemical tool to study the effect of cyanide on certain cell types. As opposed to the use of cyanide salts, these compounds would allow for the precise control over the moment and location of cyanide release. Furthermore, the masked cyanide in photo-amygdalin is considerably more cytotoxic than cyanide salts. Ultimately, photo-amygdalin compounds may find use in anticancer therapy. However, for this purpose, their potency needs to be improved, since the initial compounds reported here still require μM concentrations for cyanide phototoxicity. Also, instability and the accompanying cytotoxicity of 3°-PA and 3°-TPP in the dark so far remains a drawback, requiring the future design of new cyanogenic PPGs with superior stability. The instability of these compounds may be due to high stability of the incipient PPG-cation formed after heterolysis, as also implied by the high QY of these compounds. Having demonstrated the novel concept of photoactive cyanogenic compounds in cells, installing different inductive electron-withdrawing substituents at the PPG alpha-carbon may enhance the stability of these compounds, providing exciting opportunities for future improved versions of photo-amygdalin and applications in photopharmacology.

## Author contributions

A. M. S. performed the synthesis, photochemical measurements and conceived and performed the myoglobin test. A. G. performed the cell viability assays. G. A. performed the DFT calculations. All authors contributed to completing the manuscript. W. S. and B. L. F. supervised the project.

## Conflicts of interest

There are no conflicts to declare.

## Supplementary Material

SC-016-D5SC01248A-s001

## Data Availability

The data supporting this article have been included as part of the ESI.†
