# Supplementary material for: Photo-amygdalin: light-dependent control over hydrogen cyanide release and cytotoxicity
Source: Chem Sci. 2025 May 9;16(23):10585–94. doi: 10.1039/d5sc01248a (PMC12079188; doi:10.1039/d5sc01248a)
Supplement: SC-016-D5SC01248A-s001 [file SC-016-D5SC01248A-s001.pdf]

Supplementary Information (SI) for Chemical Science.
This journal is © The Royal Society of Chemistry 2025

Photo-Amygdalin: Light-dependent control over hydrogen cyanide release and cytotoxicity

Albert Marten Schulte, Ainoa Guinart, Georgios Alachouzos, Wiktor Szymanski, Ben L. Feringa

Contents

General remarks 2
1. Synthetic methods 3
   1.1 Synthetic scheme 3
   1.2 Experimental procedures 3
   1.3 NMR-spectra 6
2. Photochemistry 9
   General information 9
   2.1 UV-Vis absorption spectra upon irradiation 10
   2.2 ^{1}H-NMR spectra of **2°-PA** and **3°-PA** upon irradiation 12
   2.3 QY of PPG degradation 13
   2.4 HPLC-MS chromatograms of uncaging of **2°-PA** and **3°-PA** 15
   2.5 Myoglobin test for cyanide release 18
3. Hydrolytic stability 21
   3.1 UV-Vis 21
   3.2 UPLC-MS 22
4. Cell viability 30
   4.1 Cell Culture 30
   4.2 Cell viability assay 30
   4.3 ANOVA-tests 32
5. Computational data 35
   5.1 Overview of Methods and Results 35
   5.2. Optimized Geometries and XYZ Coordinates 37
6. References 59

General remarks

All reactions were performed without excluding moisture or air, unless stated otherwise. Standard Schlenk techniques were used for reactions requiring an inert atmosphere (using nitrogen as the inert gas). Reagents were purchased from commercial suppliers (Sigma-Aldrich, Combi-Blocks, TCI etc.) and used without further purification. Solvents were purchased from Boom B.V. or Sigma-Aldrich. Flash chromatography was performed on silica gel (Supelco, silica gel 60) with a particle size of 40-64 µm. TLC analysis was conducted on TLC plates with a silica gel matrix (Supelco, silica gel 60) with detection by UV-light (254 or 366 nm).

Nuclear magnetic resonance (NMR) spectra were recorded on an Agilent Technologies 400-MR (400/54 Premium Shielded) spectrometer (400 MHz for ^{1}H nucleus, 101 MHz for ^{13}C nucleus). Deuterated solvents (DMSO-d_6, $CDCl_3$ and D_2O) were purchased from Sigma-Aldrich. The chemical shift of compound resonances is given in parts per million (ppm, δ) and reported relative to the residual solvent proton or carbon resonance. All spectra were measured at ambient temperature. ^{1}H-NMR data are reported as: chemical shift, multiplicity (s = singlet, d = doublet, t = triplet, q = quartet, m = multiplet, dd = doublet of doublets, dt = doublet of triplets, dq = doublet of quartets, br = broad), coupling constants (J) given in Hz, and integration. ^{13}C-NMR spectra were conducted with proton decoupling and the chemical shifts are reported.

High resolution mass spectra (HRMS) were recorded on a Thermofisher LTQ Orbitrap XL with eluent MeOH (0.1 % TFA) and flow rate of 0.15 mL min^{-1} in positive (ACPI/ESI) mode. UV-vis spectra were recorded with an Agilent 8543 spectrophotometer. Raw data were processed using Agilent UV-vis Chemstation B.02.01 SP1, Spectragryph 1.2, OriginPro 8.5 and MS Excel.

1. Synthetic methods

1.1 Synthetic scheme

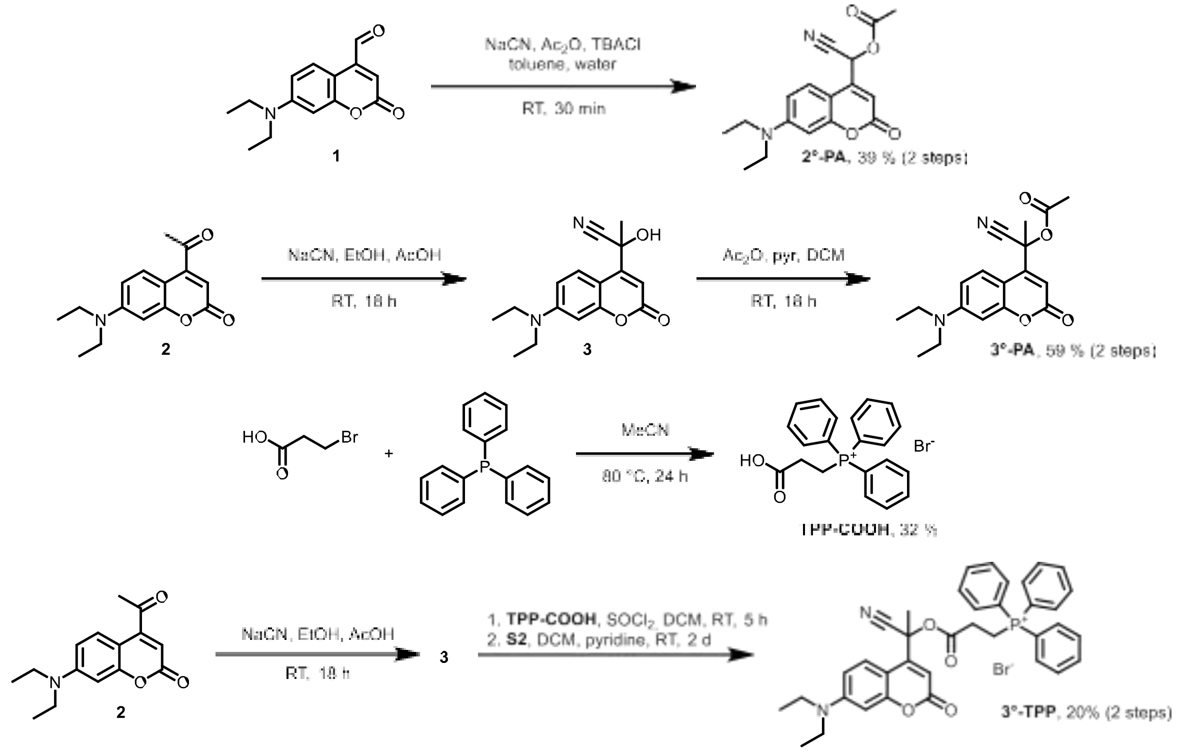

1.2 Experimental procedures

2°-PA (cyano(7-(diethylamino)-2-oxo-2H-chromen-4-yl)methyl acetate):

A one pot-procedure was used, modified from a patent.[1] Two separate solutions of aldehyde **1**[2] (100 mg, 0.41 mmol, 1.00 equiv.) and Ac_2O (46 µL, 0.49 mmol, 1.20 equiv.) were prepared in toluene (2 x 1 mL). A round bottom flask was charged with NaCN (-warning, toxic substance- 28 mg, 0.57 mmol, 1.40 equiv.) tetrabutylammonium chloride (TBACl) (11 mg, 0.04 mmol, 0.10 equiv.), water (1 mL) and toluene (2 mL). The solutions of **1** and Ac_2O in toluene were added simultaneously over a 1 min period. The mixture was stirred at RT for 30 min and then it was diluted with DCM (10 mL)> The organic layer was washed with water (10 mL) and brine (10 mL), dried over $MgSO_4$ and concentrated under reduced pressure. The crude product was purified by silica gel chromatography (pentane/EtOAc 5:1 to 3:1) to yield secondary cyanocoumarin **2°-PA** as a yellow solid (75 mg, 59 %). **^{1}H-NMR** (400 MHz, $CDCl_3$) δ 7.38 (d, *J* = 9.1 Hz, 1H), 6.63 (dd, *J* = 9.1, 2.3 Hz, 1H), 6.56 – 6.51 (m, 2H), 6.31 (s, 1H), 3.43 (q, *J* = 7.1 Hz, 4H), 2.25 (s, 3H), 1.22 (t, *J* = 7.1 Hz, 6H). **^{13}C-NMR** (101 MHz, $CDCl_3$) δ 168.5, 160.9, 156.9, 151.4, 144.2, 124.6, 114.4, 109.3, 108.7, 104.4, 98.2, 59.0, 45.0, 20.5, 12.6. **HRMS** (ESI): calc. for $C_{17}H_{18}N_2O_4H^+$ (M+H): 315.1339; found: 315.1339

3°-PA (1-cyano-1-(7-(diethylamino)-2-oxo-2H-chromen-4-yl)ethyl acetate):

Ketone **2**[3] (94 mg, 0.36 mmol, 1.00 equiv.) was dissolved in a mixture of EtOH (1.4 mL) and AcOH (0.6 mL). NaCN (-warning, toxic substance- 231 mg, 1.71 mmol, 13.00 equiv.) was added and the mixture was stirred at RT for 18 h. Subsequently, the mixture was diluted with water (20 mL) and extracted with EtOAc (2 x 20 mL). The combined organic layers were washed with water (2 x) and brine, dried over $MgSO_4$ and concentrated under reduced pressure (temperature of the rotary evaporator water bath was set at 35 °C). The crude cyanohydrin was dissolved in DCM (3.6 mL) and Ac_2O (0.17 mL, 1.82 mmol, 5.00 equiv.) and pyridine (32 µL, 0.40 mmol, 1.10 eq.) were added. The mixture was stirred at RT for 2 h, after which additional portions of Ac_2O (0.17 mL, 1.82 mmol, 5.00 equiv.) and pyridine (0.15 mL, 1.82 mmol, 5.00 equiv.) were added. After stirring an additional 16 h at RT in the dark, the mixture was diluted with DCM (20 mL), washed with 1 M HCL (20 mL), brine, dried over $MgSO_4$, and concentrated under reduced pressure. The crude product was purified by silica gel chromatography (a column using DCM/acetone 98:2 to 96:4, followed by another column using Pentane/EtOAC (4:1 to 3:1), to yield tertiary cyanocoumarin **2** as a yellow solid (70 mg, 59 %). **^{1}H-NMR** (400 MHz, $CDCl_3$) δ 7.68 (d, *J* = 9.3 Hz, 1H), 6.57 (dd, *J* = 9.3, 2.8 Hz, 1H), 6.53 (d, *J* = 2.7 Hz, 1H), 6.42 (s, 1H), 3.41 (q, *J* = 7.2 Hz, 4H), 2.17 (s, 3H), 2.15 (s, 3H), 1.21 (t, *J* = 7.1 Hz, 6H). **^{13}C-NMR** (101 MHz, $CDCl_3$) δ 168.0, 161.1, 157.2, 150.7, 149.6, 125.6, 117.0, 108.9, 107.5, 103.3, 98.6, 72.7, 44.9, 27.2, 20.8, 12.6. **HRMS** (ESI): calc. for $C_{18}H_{20}N_2O_4H^+$ (M+H): 329.1496; found: 329.1496

TPP-COOH ((2-carboxyethyl)triphenylphosphonium bromide):

TPP-OAc was prepared according to a literature procedure.[4] **^{1}H-NMR** (400 MHz, $CDCl_3$) δ 7.87 – 7.63 (m, 15H), 3.74 (dt, *J* = 12.6, 7.6 Hz, 2H), 3.03 (dt, *J* = 14.9, 7.5 Hz, 2H). **^{13}C-NMR** (101 MHz, $CDCl_3$) δ 170.7 (d, *J* = 13.6 Hz), 135.0 (d, *J* = 3.0 Hz), 133.2 (d, *J* = 10.1 Hz), 130.2 (d, *J* = 12.6 Hz), 117.0 (d, *J* = 86.6 Hz), 27.4 (d, *J* = 2.6 Hz), 18.2 (d, *J* = 55.0 Hz). **^{31}P-NMR** (162 MHz, $CDCl_3$) δ 24.64. Spectra in accordance with literature.[4]

3°-TPP (3-(1-cyano-1-(7-(diethylamino)-2-oxo-2H-chromen-4-yl)ethoxy)-3-oxopropyl)triphenylphosphonium bromide:

To a suspension of ketone **2**[3] (75 mg, 0.29 mmol, 1.00 equiv.) in EtOH (2 mL) and AcOH (0.8 mL) was added NaCN (184 mg, 3.76 mmol, 13.00 equiv.). The mixture was stirred at RT for 18 h, diluted with EtOAc (10 mL), and washed with water (2 x 10 mL) and brine (10 mL). The organic layer was dried over $MgSO_4$, and concentrated under reduced pressure (the rotary evaporator water bath was set at 35 °C). The residue was passed through a silica plug (DCM/acetone 95:5) to yield the crude cyanohydrin **3**.

Subsequently, **TPP-OAc** (86 mg, 0.21 mmol, 1.00 equiv.) was dissolved in dry DCM (2 mL) under nitrogen atmosphere, and $SOCl_2$ (22 µL, 0.31 mmol, 1.48 equiv.) was added. The mixture was stirred at RT for 5 min, and a solution of **3** (73 mg, 0.26 mmol, 1.23 equiv.) in dry DCM (1 mL) was added, followed by dry pyridine (21 µL, 0.26 mmol, 1.23 equiv.). The mixture was stirred at RT for 2 d, and concentrated under reduced pressure. The crude residue was purified by silica gel chromatography (DCM/MeOH 97:2 to

92:8, followed by another column acetone/MeOH (99:1 to 92:8) to yield **3°-TPP** as an orange solid (40 mg, 20 % over 2 steps). **^{1}H-NMR** (400 MHz, $CDCl_3$) δ 7.82 – 7.75 (m, 9H), 7.69 – 7.63 (m, 6H), 7.62 (d, *J* = 9.4 Hz, 1H), 6.71 (dd, *J* = 9.3, 2.7 Hz, 1H), 6.48 (d, *J* = 2.7 Hz, 1H), 6.32 (s, 1H), 4.34 (dddd, *J* = 16.5, 12.5, 8.3, 4.0 Hz, 1H), 3.98 (dddd, *J* = 16.3, 12.7, 8.7, 4.0 Hz, 1H), 3.53 – 3.16 (m, 6H), 2.07 (s, 3H), 1.20 (t, *J* = 7.1 Hz, 6H). **^{13}C-NMR** (101 MHz, $CDCl_3$) δ 168.9 (d, *J* = 5.9 Hz), 161.0, 157.1, 150.9, 148.9, 135.5 (d, *J* = 3.0 Hz), 133.9 (d, *J* = 10.0 Hz), 130.7 (d, *J* = 12.7 Hz), 126.1, 117.8 (d, *J* = 86.5 Hz), 116.4, 110.1, 107.3, 102.6, 98.2, 74.4, 44.9, 27.8 (d, *J* = 3.9 Hz), 27.1, 18.2 (d, *J* = 54.7 Hz), 12.6. **^{31}P-NMR** (162 MHz, $CDCl_3$) δ 25.24. **HRMS** (ESI): calc. for $C_{37}H_{36}N_2O_4P^+$ (M+): 603.2407; found: 603.2407

1.3 NMR-spectra

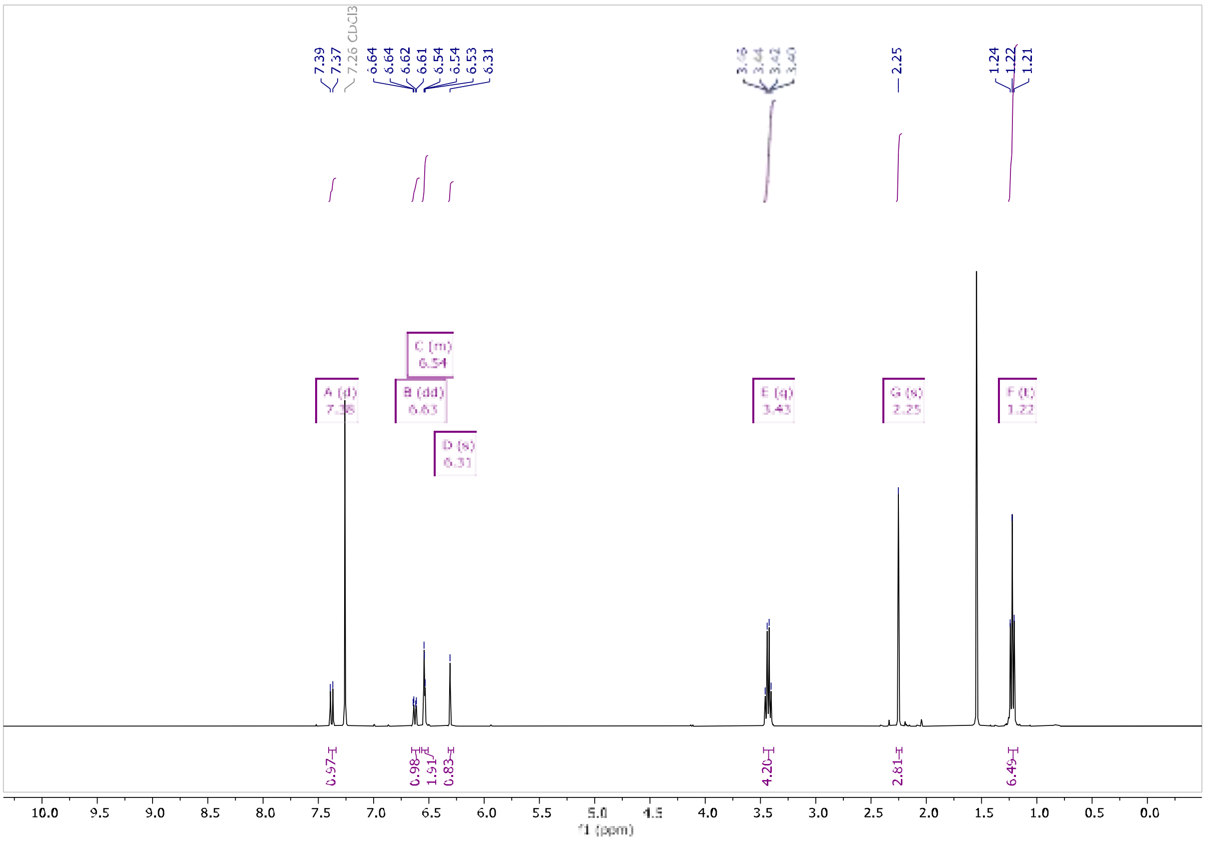

Figure S1. ^{1}H-NMR spectrum of compound **2°-PA** ($CDCl_3$).

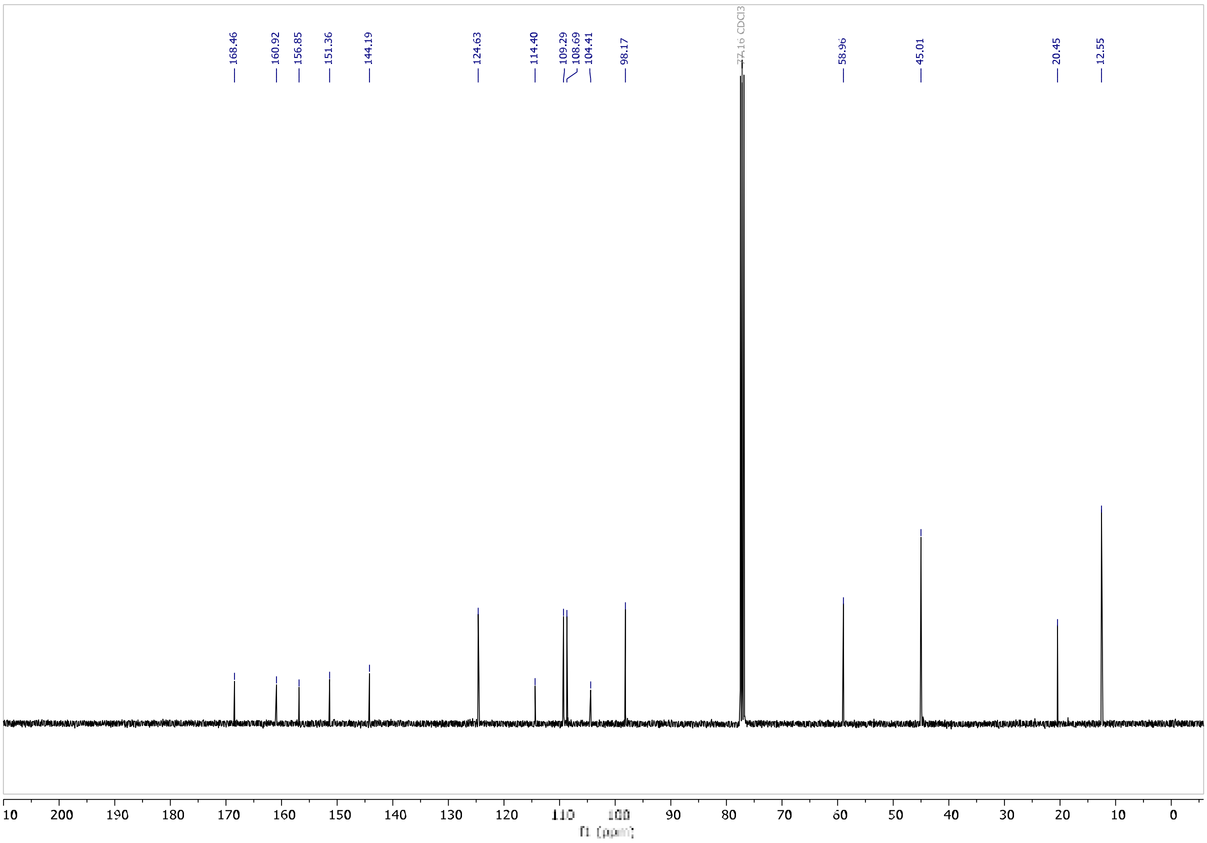

Figure S 2. ^{13}C-NMR spectrum of compound **2°-PA** ($CDCl_3$).

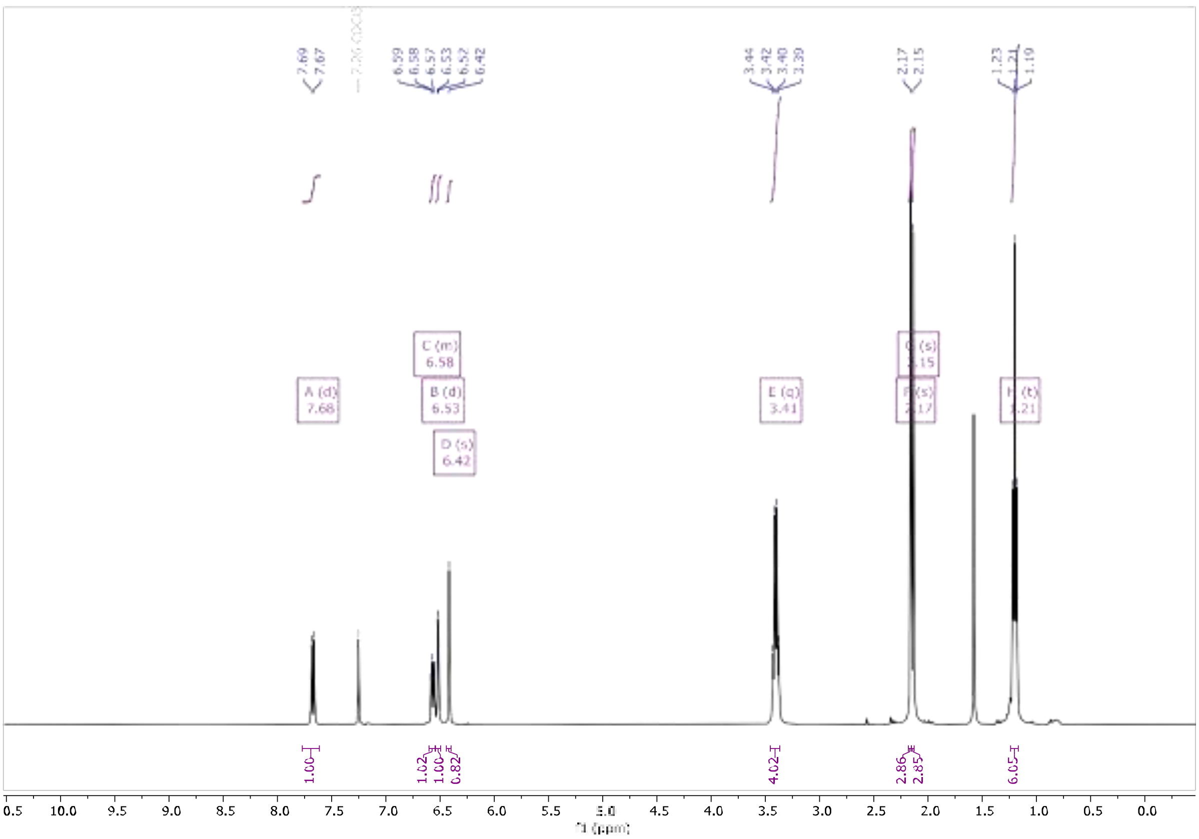

Figure S3. ^{1}H-NMR spectrum of compound **3°-PA** (CDCl$_3$).

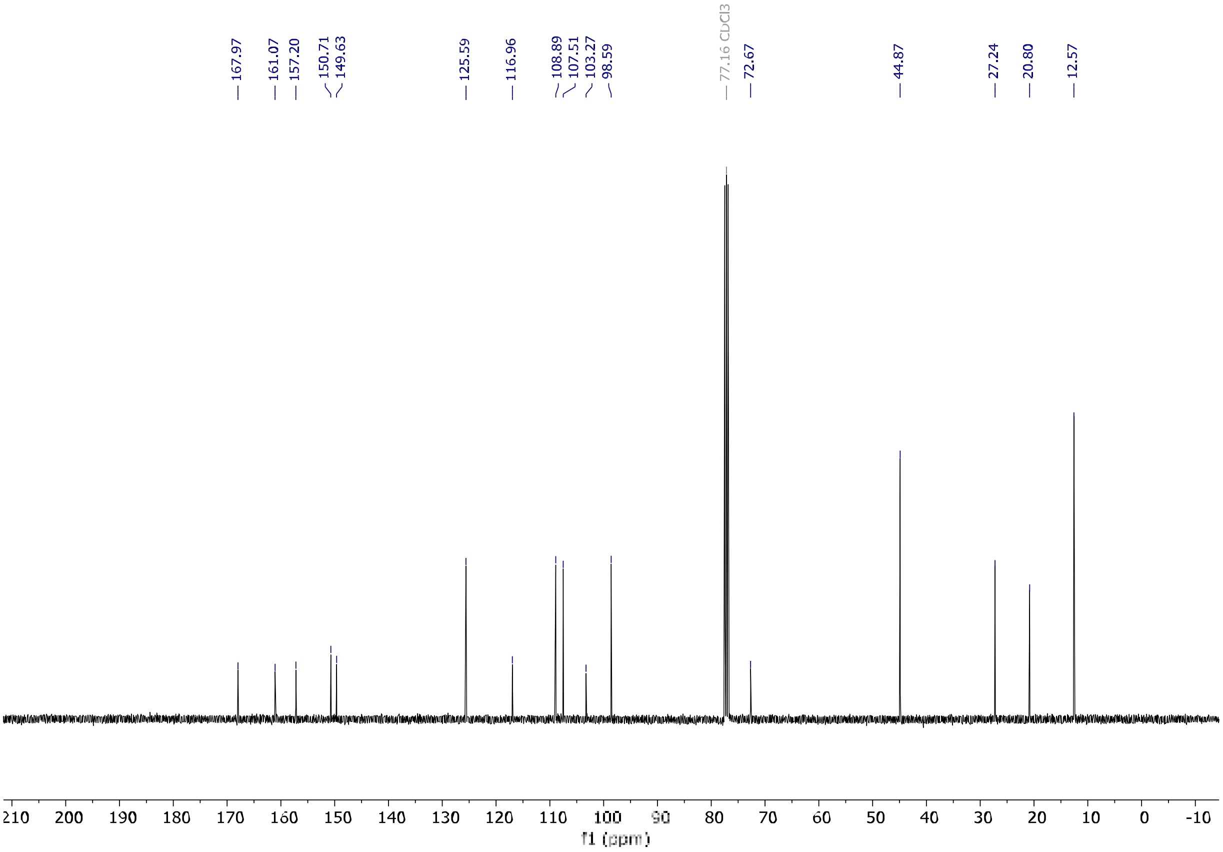

Figure S4. ^{13}C-NMR spectrum of compound **3°-PA** (CDCl$_3$).

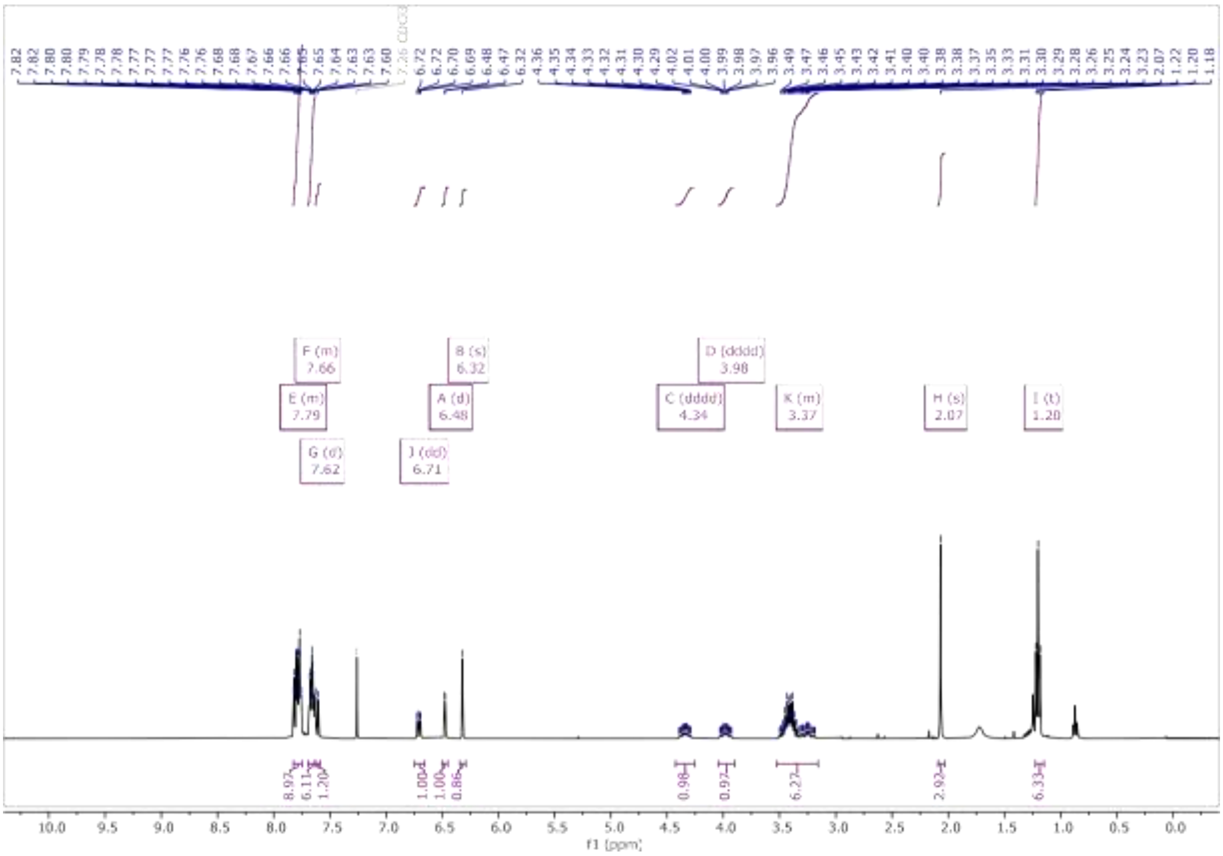

Figure S5. ^{1}H-NMR spectrum of compound **3°-TPP** ($CDCl_3$).

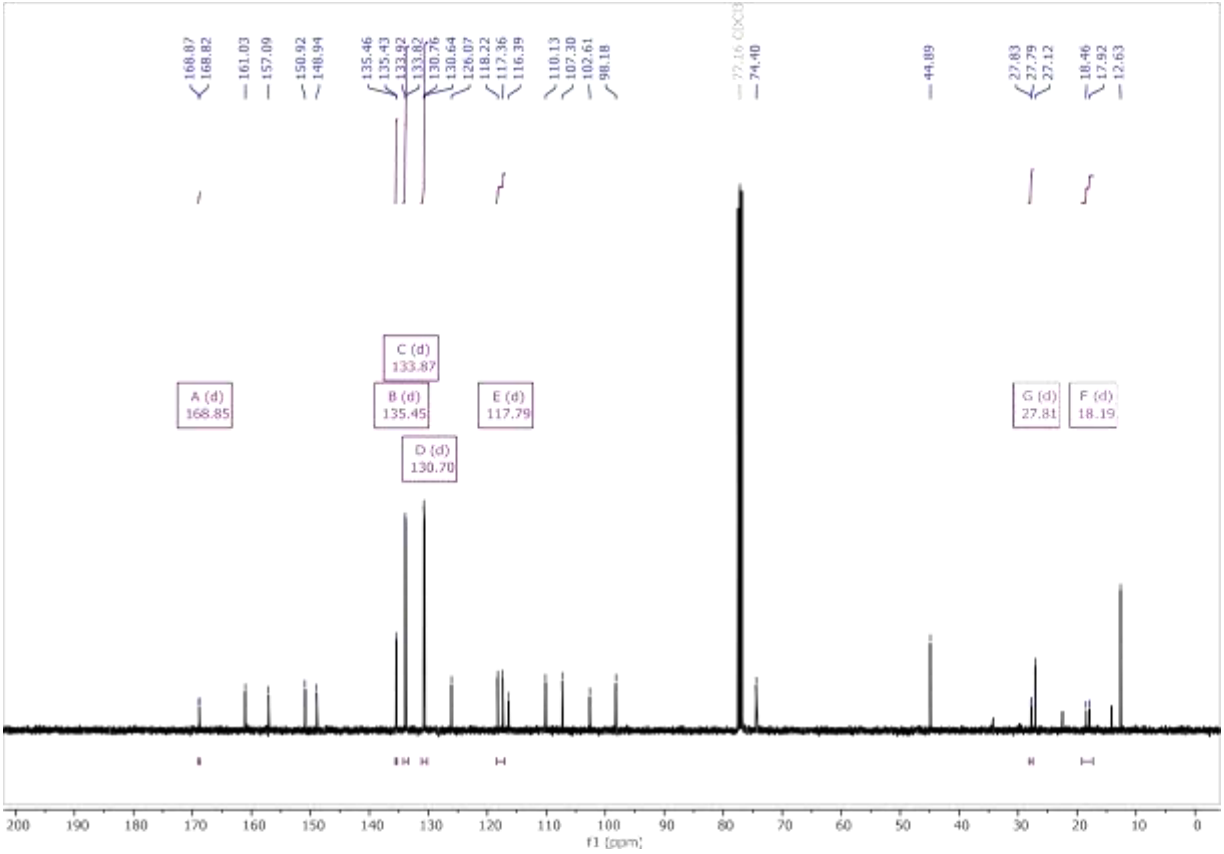

Figure S6. ^{13}C-NMR spectrum of compound **3°-TPP** ($CDCl_3$).

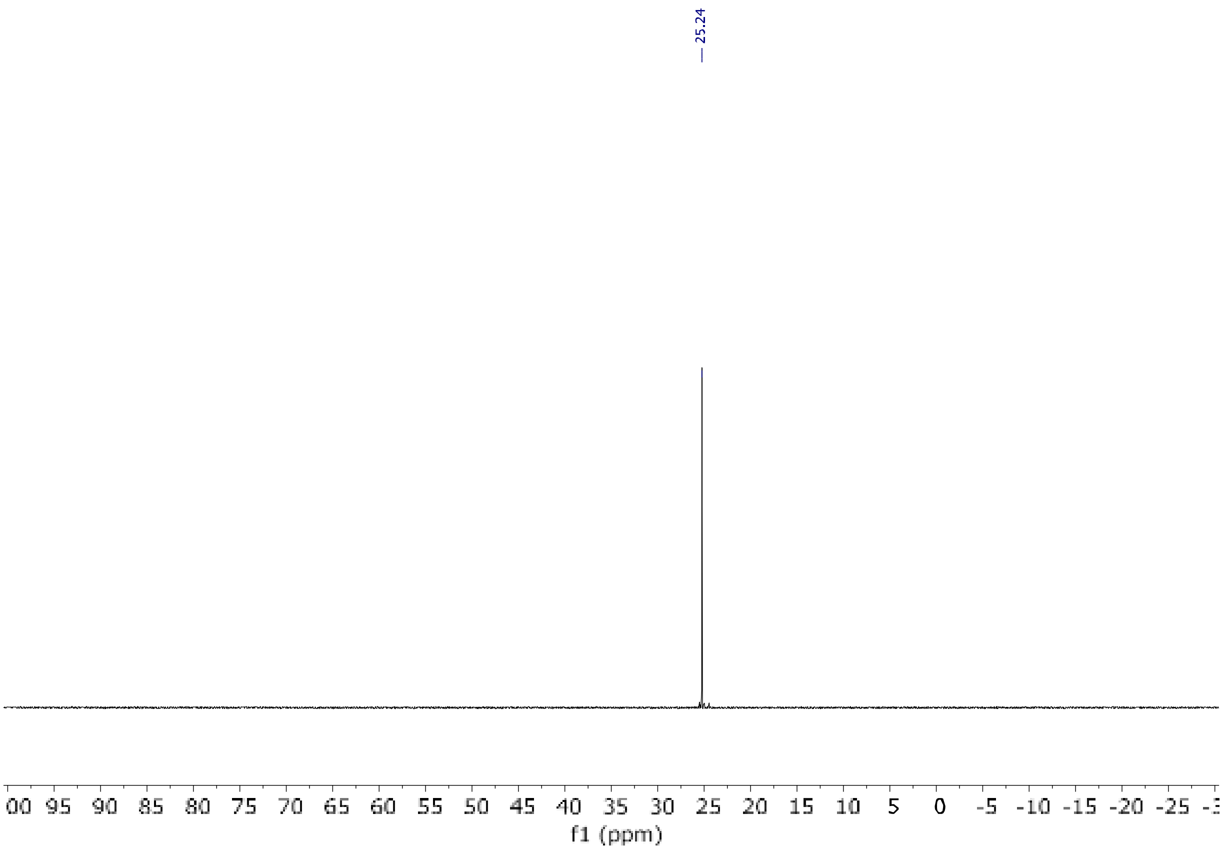

Figure S7. ^{31}P-NMR spectrum of compound **3°-TPP** ($CDCl_3$).

2. Photochemistry

General information

A Prizmatix FC6-LED Multi channel fiber coupled LED source was used. For irradiation of the coumarin PPGs, the 390 nm channel was used which consists of a LED with a peak wavelength at 389.7 nm, FWHM 14.3 nm and -depending on when the measurement was taken- a photon flux as reported in the caption of every figure that reports a QY. 2 mL solutions were irradiated in a quartz cuvette (10 x 10 mm) from the side.

2.1 UV-Vis absorption spectra upon irradiation

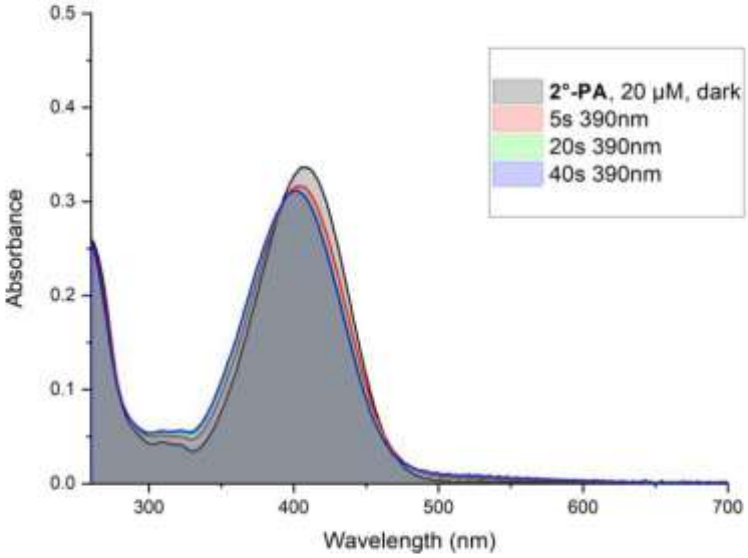

Figure S8. UV-Vis absorption spectra of **2°-PA** (20 µM, 99:1 PBS-buffer pH 7.3/DMSO, 25 °C) in the dark (black line) and upon irradiation for the times indicated (colored lines).

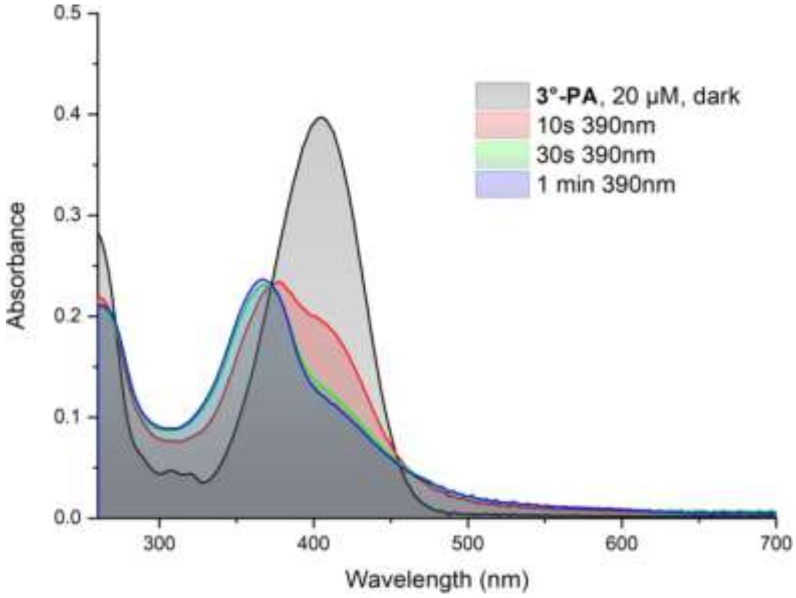

Figure S9. UV-Vis absorption spectra of **3°-PA** (20 µM, 99:1 PBS-buffer pH 7.3/DMSO, 25 °C) in the dark (black line) and upon irradiation for the times indicated (colored lines).

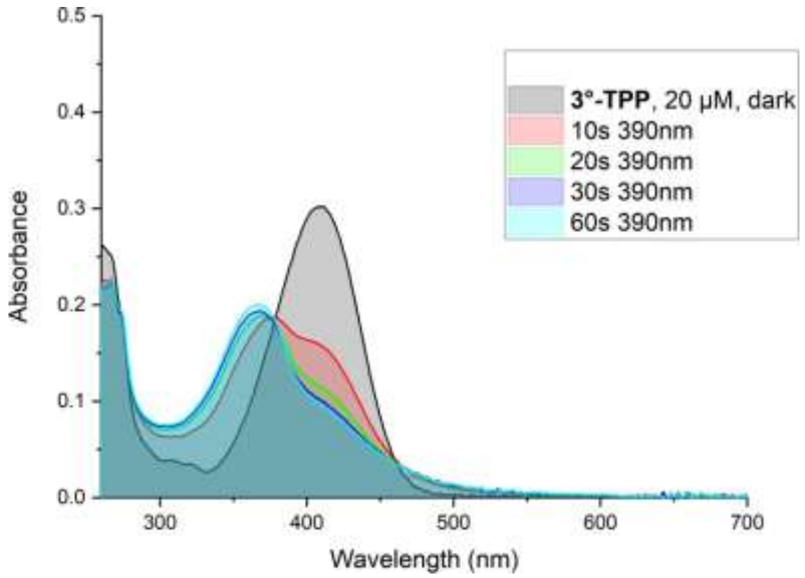

Figure S10. UV-Vis absorption spectra of **3°-TPP** (20 µM, 99:1 PBS-buffer pH 7.3/DMSO, 25 °C) in the dark (black line) and upon irradiation for the times indicated (colored lines).

2.2 ^{1}H-NMR spectra of 2°-PA and 3°-PA upon irradiation

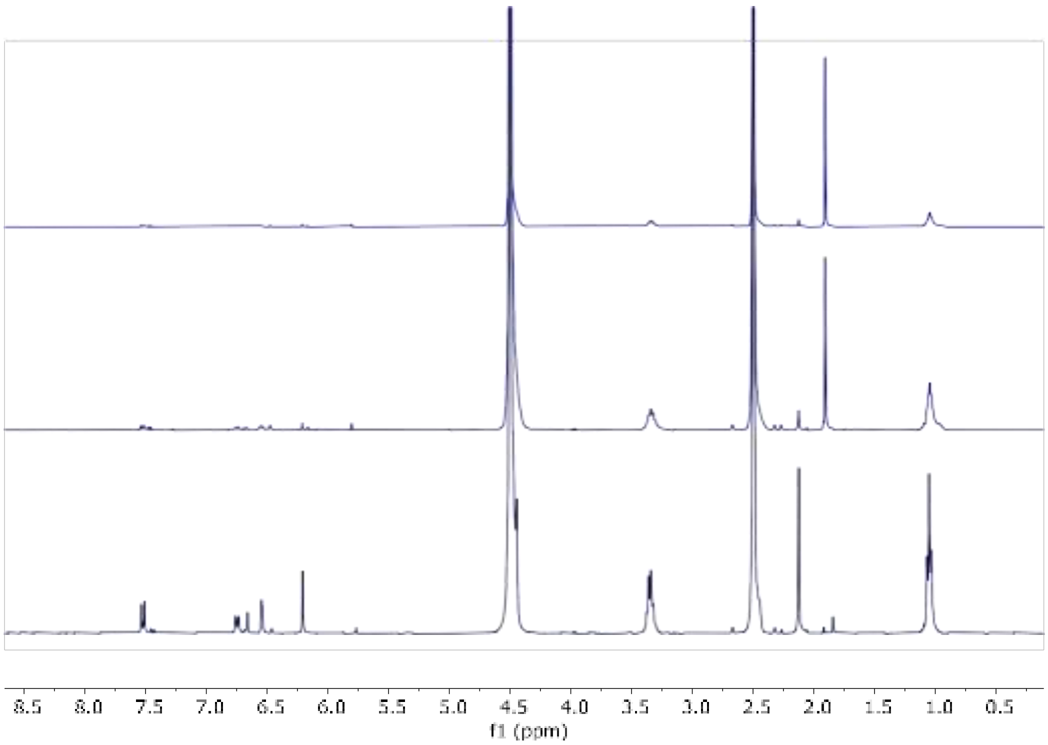

Figure S11. ^{1}H-NMR spectra of compound **2°-PA** (2 mM DMSO-d_6/D_2O 1:1) in the dark (bottom spectrum) and after irradiation with blue light (λ_{max} = 400 nm) for 5 min (middle spectrum). The release of AcOH was confirmed through a spike with AcOH (top spectrum, signal at ~1.9 ppm).

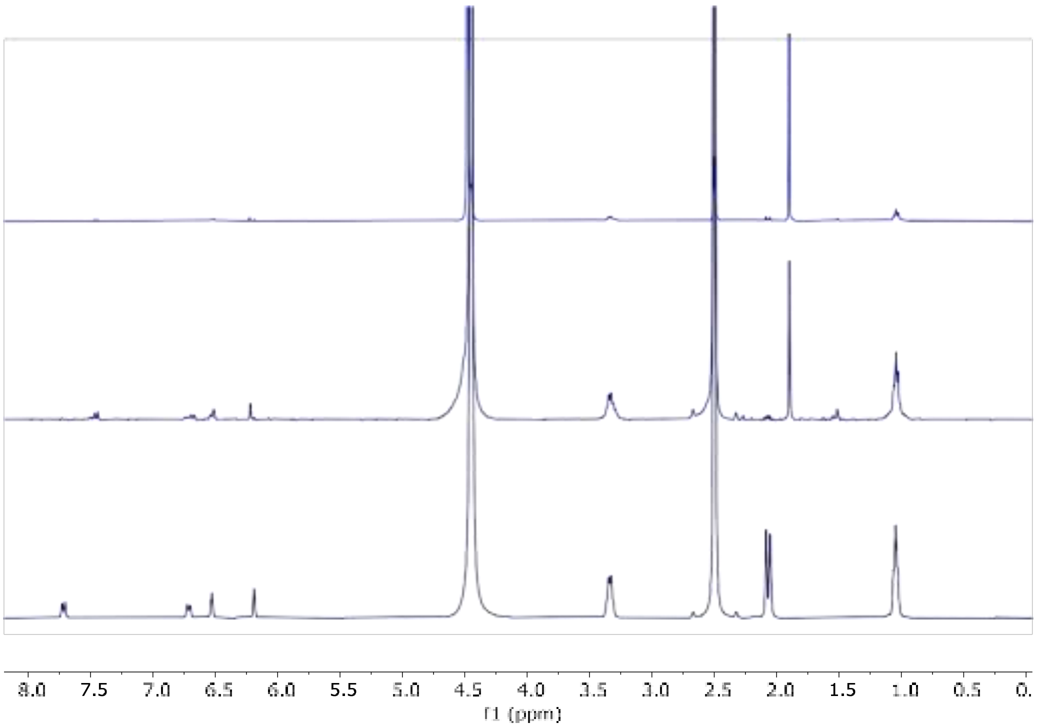

Figure S12. ^{1}H-NMR spectra of compound **3°-PA** (2 mM DMSO-d_6/D_2O 1:1) in the dark (bottom spectrum) and after irradiation with blue light (λ_{max} = 400 nm) for 10 min (middle spectrum). The release of AcOH was confirmed through a spike with AcOH (top spectrum, signal at ~1.9 ppm).

2.3 QY of PPG degradation

The QY of PPG degradation was determined using the following formula:

$$\frac{conversion\ rate\ (s^{-1}) \times concentration\ (mM) \times volume\ (L)}{corrected\ photon\ flux\ (mmol \times s^{-1})}$$

The normalized conversion rate was determined by UV-vis spectroscopy through following PPG consumption at a fixed wavelength upon irradiation (λ = 390 nm). The average rate over the first 10 % of conversion was determined through fitting a trend-line and multiplied by the concentration and volume of the sample to reach a PPG consumption rate in mmol · s^{-1}. This rate was divided by the photon flux at λ = 390 nm (7.048 · 10^{-6} mmol · s^{-1}, determined by ferrioxalate actinometry[5]) that was corrected for the specific absorbance of each sample at the irradiation wavelength (A ≈ 1, values reported, λ = 390 nm). All measurements were performed in triplicate and averages and standard deviations are reported for all QYs.

To accurately measure the QY, we first start recording absorption spectra before turning on the LED irradiation source and starting the photochemical reaction. This way, we capture the initial rate of uncaging and don't miss it through first turning on the LED and afterwards start recording spectra. A result of this technique is that we always have to omit the first spectrum that's recorded by our spectrophotometer because the irradiation LED was turned on somewhere between this first measurement and the second measurement. This means that substrate conversion between measurement 1 and 2 will be less than the conversion between the later measurements, because the LED was only turned on for part of the time between these first 2 measurements. If we didn't omit the first measurement, our QY values will be slightly off. Because of this, the conversion plots always start slightly above zero, caused by the conversion that is achieved between measurement 1 and measurement 2.

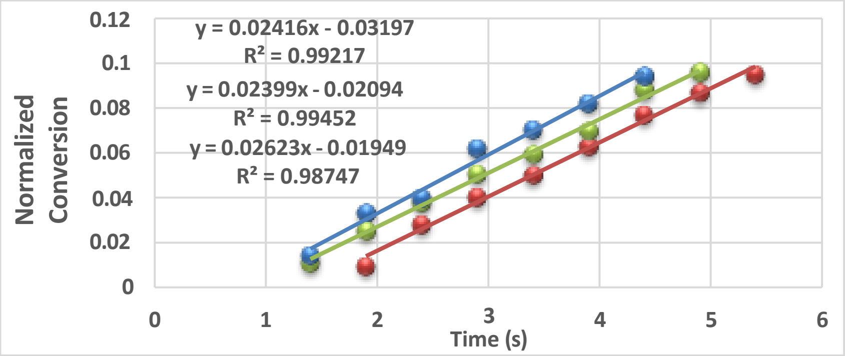

water/MeCN 97:3	2°-PA	rate	A_{390}	QY
	#1	0.02416	0.79	0.450
	#2	0.02399	0.78	0.449
	#3	0.02623	0.82	0.482
			**Av. QY**	**0.460**
			**SD**	**0.016**

Figure S13. Normalized conversion of compound **2°-PA** (55 µM, 2 mL water/MeCN 97:3, 25 °C) (y-axis) vs time (x-axis). Shown are the measurements taken after the start of irradiation (λ = 390 nm). A linear trendline allowed for the determination of the average rate over the first 10% of PPG consumption. Conversion followed at λ = 320 nm (photon flux 7.048*10^{-6} mmol/s). Normalization of the absorbance values was performed from data collection start (t=0) to the maximum achieved absorbance value at full deprotection.

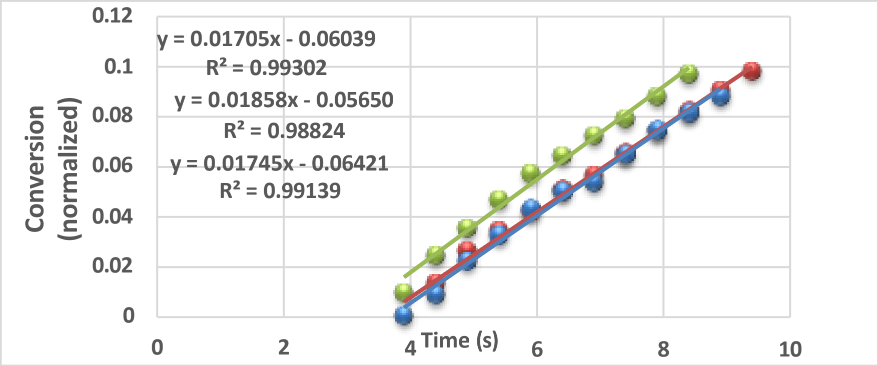

water/MeCN 97:3	3°-PA	rate	A_{390}	QY
	#1	0.01705	0.83	0.312
	#2	0.01858	0.82	0.342
	#3	0.01745	0.84	0.318
			**Av. QY**	**0.324**
			**SD**	**0.013**

Figure S14. Normalized conversion of compound **3°-PA** (55 µM, 2 mL water/MeCN 97:3, 25 °C) (y-axis) vs time (x-axis). Shown are the measurements taken after the start of irradiation (λ = 390 nm). A linear trendline allowed for the determination of the average rate over the first 10% of PPG consumption. Conversion followed at λ = 320 nm (photon flux 7.048*10^{-6} mmol/s). Normalization of the absorbance values was performed from data collection start (t=0) to the maximum achieved absorbance value at full deprotection.

2.4 HPLC-MS chromatograms of uncaging of 2°-PA and 3°-PA

Solutions of **2°-PA** and **3°-PA** (20 µM, 2 mL, 99:1 water/MeCN) were irradiated in a quartz-cuvette with violet light (λ = 390 nm) for 1 min. HPLC-MS samples were taken before and after irradiation.

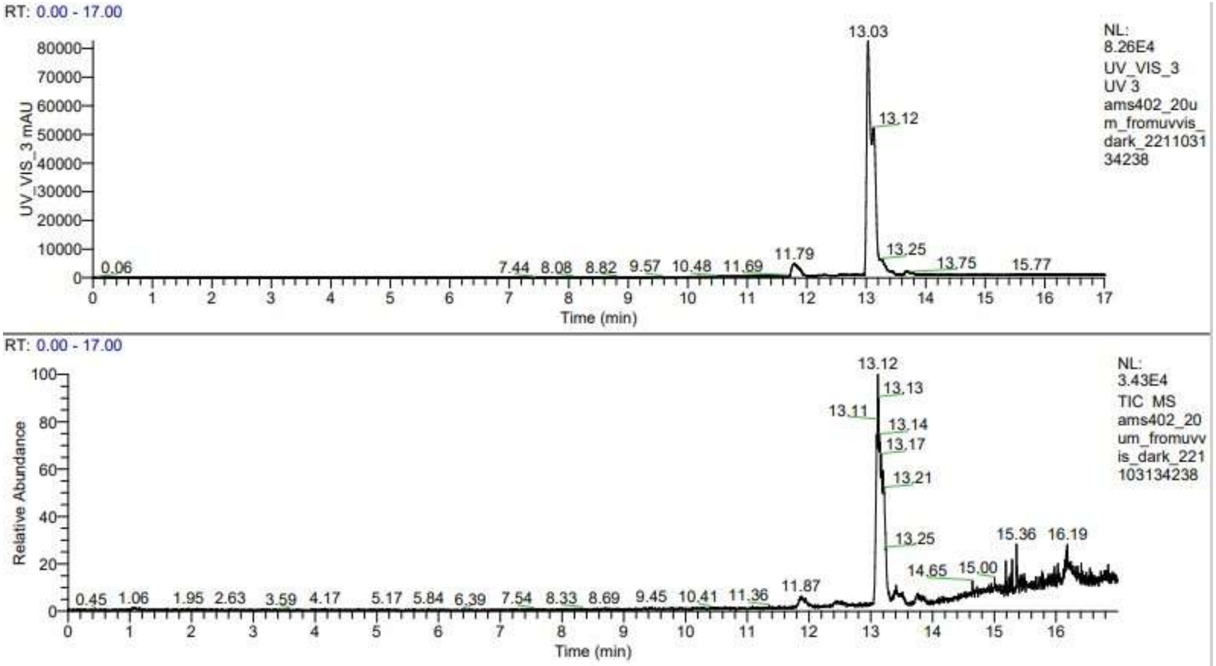

Figure S15. HPLC-MS chromatograms of a solution of **2°-PA** (20 µM 99:1 water/MeCN) before irradiation. Shown are the UV-Vis -trace (λ = 390 nm, top) and TIC-trace (bottom). The peak at 13.1 min has an m/z value of 315. Due to the hydrolytic instability of **2°-PA**, other peaks can be seen.

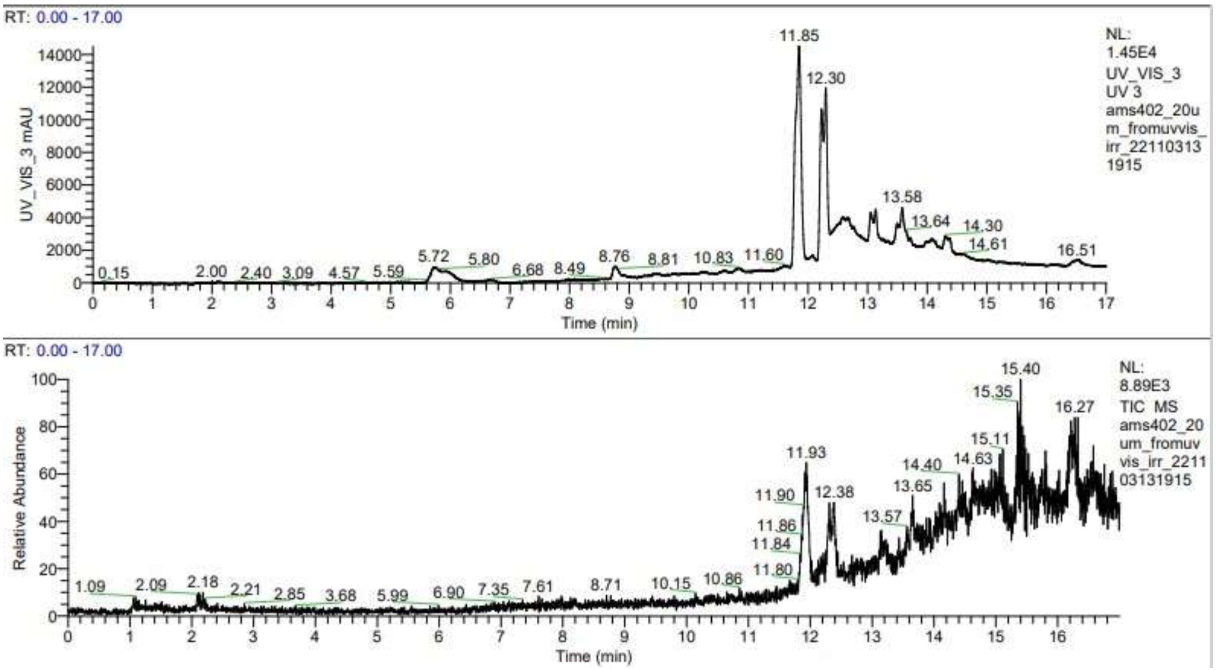

Figure S16. HPLC-MS chromatograms of a solution of **2°-PA** (20 µM 99:1 water/MeCN) after irradiation (1 min, λ = 390 nm). Shown are the UV-Vis trace (λ = 390 nm, top) and TIC-trace (bottom).

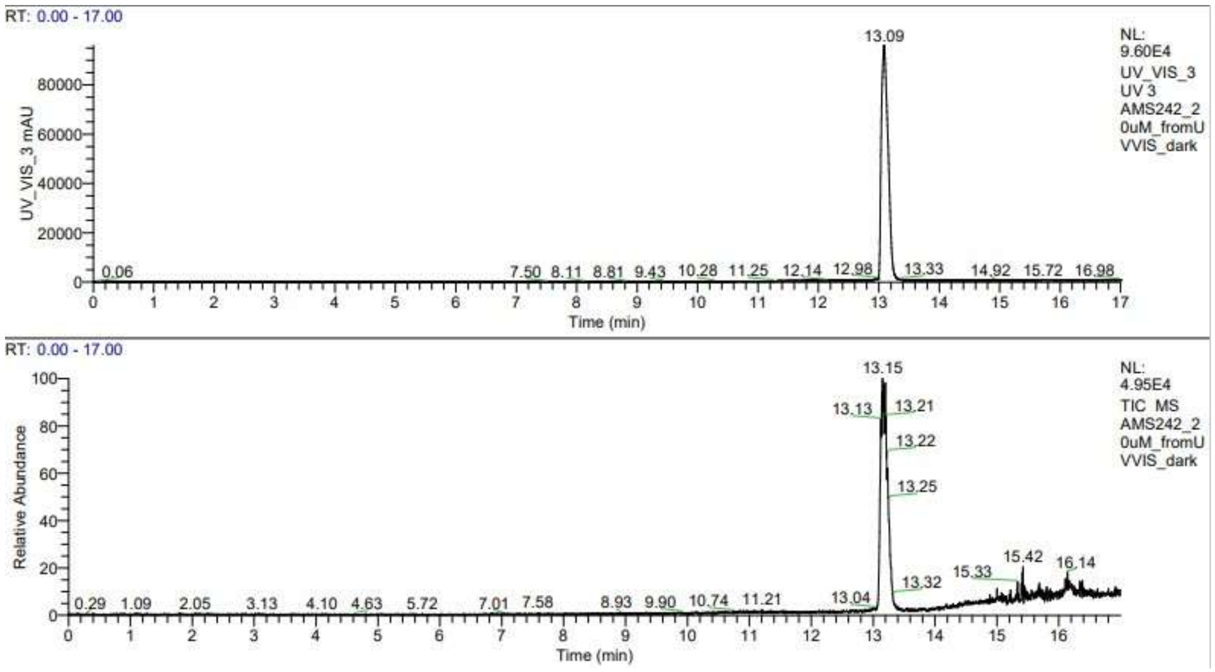

Figure S17. HPLC-MS chromatograms of a solution of **3°-PA** (20 µM 99:1 water/MeCN) before irradiation. Shown are the UV-Vis trace (λ = 390 nm, top) and TIC-trace (bottom). The peak at 13.1 min has an m/z value of 329.

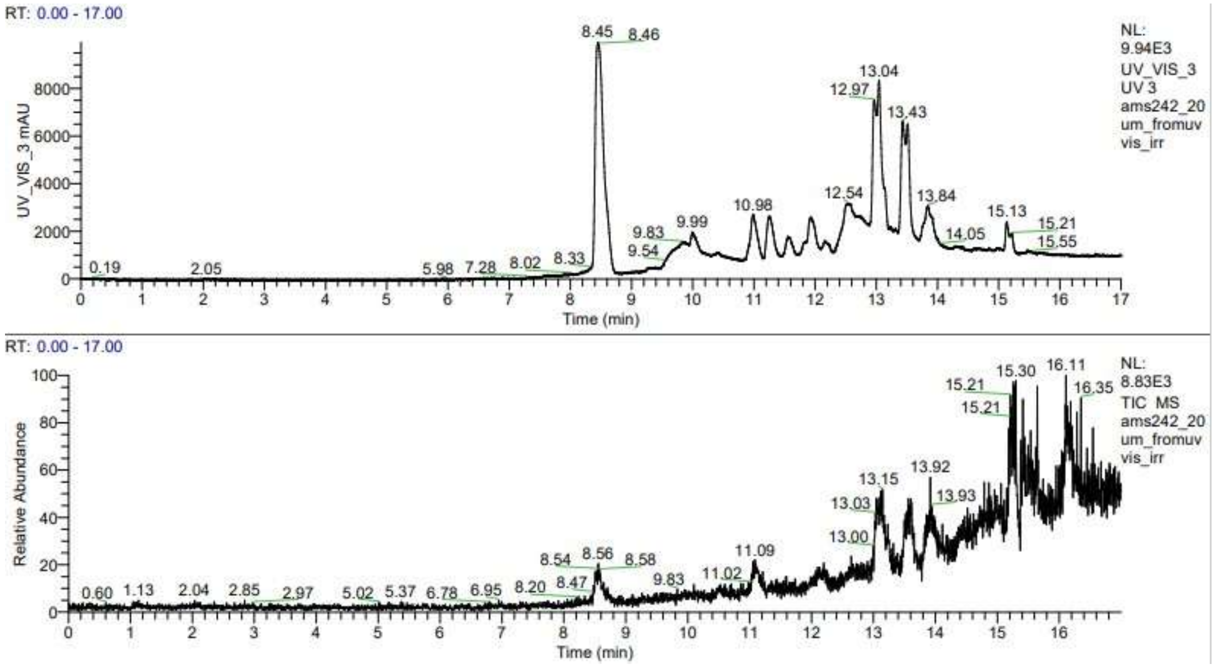

Figure S18. HPLC-MS chromatograms of a solution of **3°-PA** (20 µM 99:1 water/MeCN) after irradiation (1 min, λ = 390 nm). Shown are the UV-Vis-trace (λ = 390 nm, top) and TIC-trace (bottom).

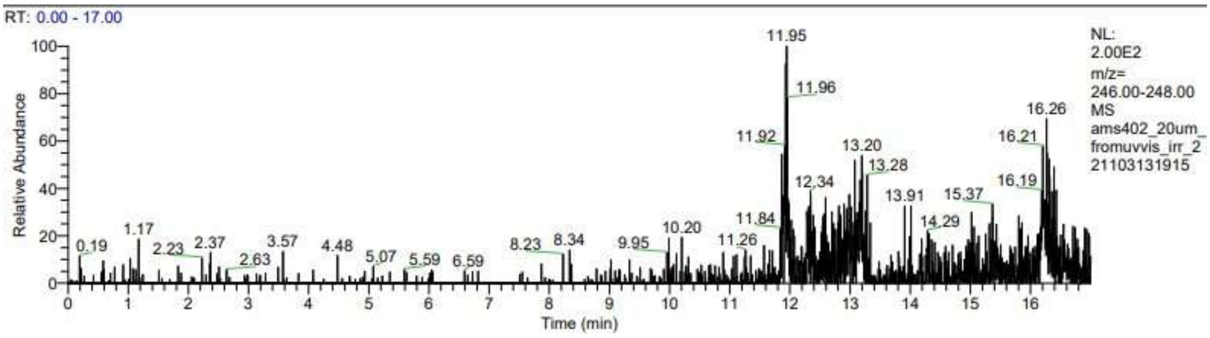

Figure S19. Mass-scan of the irradiated solution of **2°-PA** scanning for the mass of the aldehyde (m/z = 246).

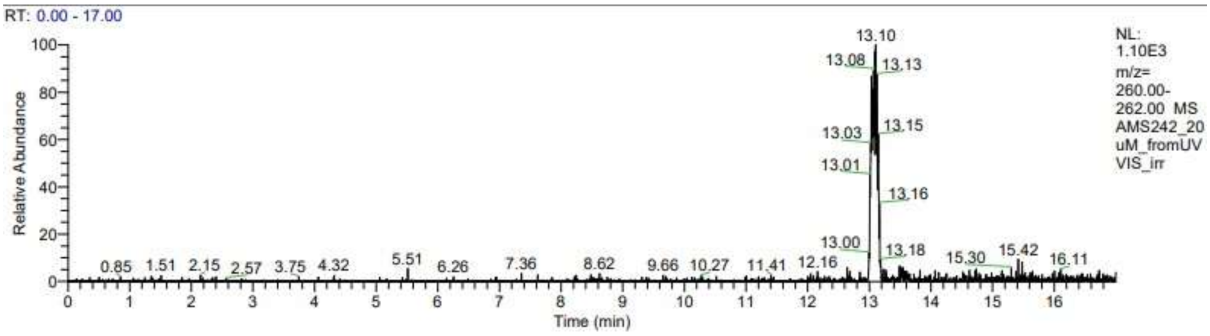

Figure S20. Mass-scan of the irradiated solution of **3°-PA** scanning for the mass of the ketone (m/z = 260).

2.5 Myoglobin test for cyanide release

A myoglobin test was developed to detect the released cyanide. The heme group in Myoglobin can bind cyanide, resulting in a bathochromic shift in its absorption spectrum.[6] Initially, this known effect of cyanide on the absorption spectrum of myoglobin was replicated in a procedure using NaCN:

To a solution of myoglobin (1.5 µM) in PBS-buffer (137 mM NaCl, 2.7 mM KCl, 10 mM Na_2HPO_4, 1.8 mM KH_2PO_4, pH 7.3, 2 mL) was added NaCN (40 µM) and the solution was incubated at 25 °C for 10 min. After this time, a clear bathochromic shift in the absorption spectrum was observed, associated with the formation of cyano-myoglobin (Figure S21).[6,7]

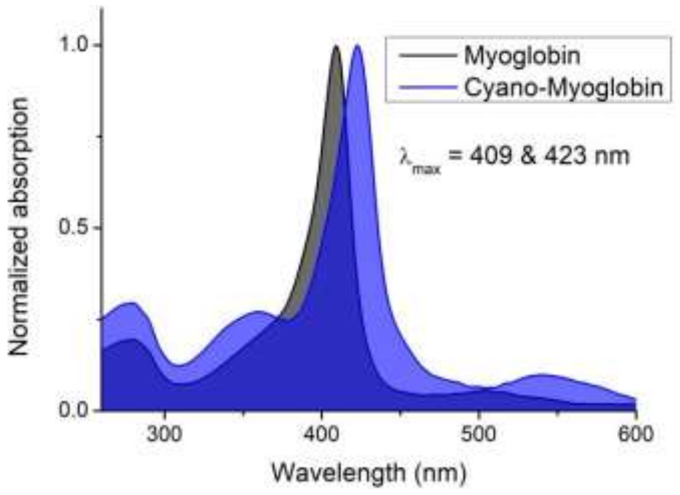

Figure S21. UV-Vis absorption spectra of Myoglobin (1.5 µM) in PBS-buffer (pH 7.3) at 25 °C in the absence or presence of cyanide (40 µM). All displayed spectra recorded after incubation at 25 °C for 10 minutes.

The following procedure was used for the cyanide detection of the Coumarin PPGs:

A solution of the coumarin PPG (50 µM, PBS-buffer pH 7.3, containing 2.5 % DMSO) at 25 °C was either irradiated (λ_{max} = 390 nm, 1 min for **2°-PA**, **3°-PA** and **3°-TPP**, 2 min for **4**) or incubated in the dark (1 min for **2°-PA**, **3°-PA** and **3°-TPP**, 2 min for **4**). Subsequently, the solution was blanked in the UV-Vis spectrophotometer and myoglobin (800 µM stock solution in PBS, 3.75 µL, 1.5 µM) was added. The resulting mixtures were incubated at 25 °C for 10 min and absorption spectra were recorded.

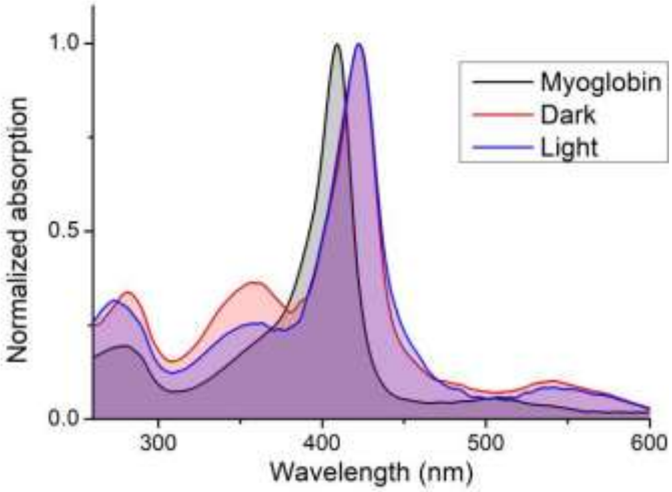

Figure S22. UV-Vis absorption spectra of Myoglobin (1.5 µM) in PBS-buffer (pH 7.3) at 25 °C in the presence of **2°-PA** (50 µM, blanked), that was either irradiated (‘light’, λ_{max} = 390 nm, 1 min) or kept in the dark for 1 min (‘dark’). Also shown is the absorption spectrum of myoglobin as reference. All displayed spectra recorded after incubation at 25 °C for 10 minutes.

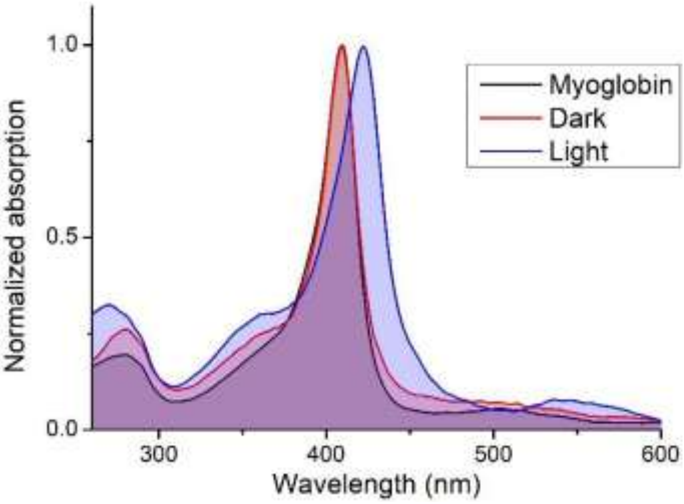

Figure S23. UV-Vis absorption spectra of Myoglobin (1.5 µM) in PBS-buffer (pH 7.3) at 25 °C in the presence of **3°-PA** (50 µM, blanked), that was either irradiated (‘light’, λ_{max} = 390 nm, 1 min) or kept in the dark for 1 min (‘dark’). Also shown is the absorption spectrum of myoglobin as reference. All displayed spectra recorded after incubation at 25 °C for 10 minutes.

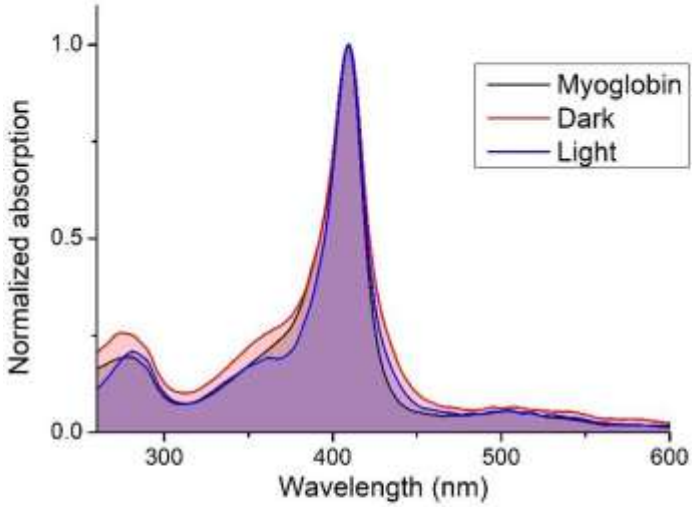

Figure S24. UV-Vis absorption spectra of Myoglobin (1.5 µM) in PBS-buffer (pH 7.3) at 25 °C in the presence of **4** (50 µM, blanked), that was either irradiated ('light', λ_{max} = 390 nm, 2 min) or kept in the dark for 2 min ('dark'). Also shown is the absorption spectrum of myoglobin as reference. All displayed spectra recorded after incubation at 25 °C for 10 minutes.

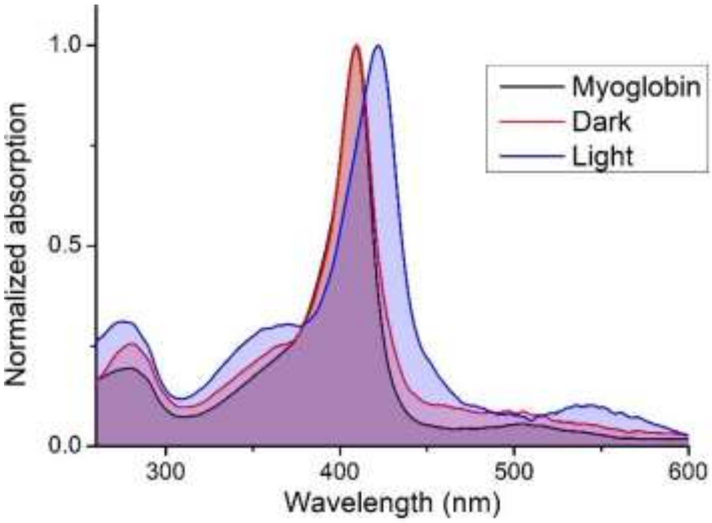

Figure S25. UV-Vis absorption spectra of Myoglobin (1.5 µM) in PBS-buffer (pH 7.3) at 25 °C in the presence of **3°-TPP** (50 µM, blanked), that was either irradiated ('light', λ_{max} = 390 nm, 1 min) or kept in the dark for 1 min ('dark'). Also shown is the absorption spectrum of myoglobin as reference. All displayed spectra recorded after incubation at 25 °C for 10 minutes.

3. Hydrolytic stability

3.1 UV-Vis

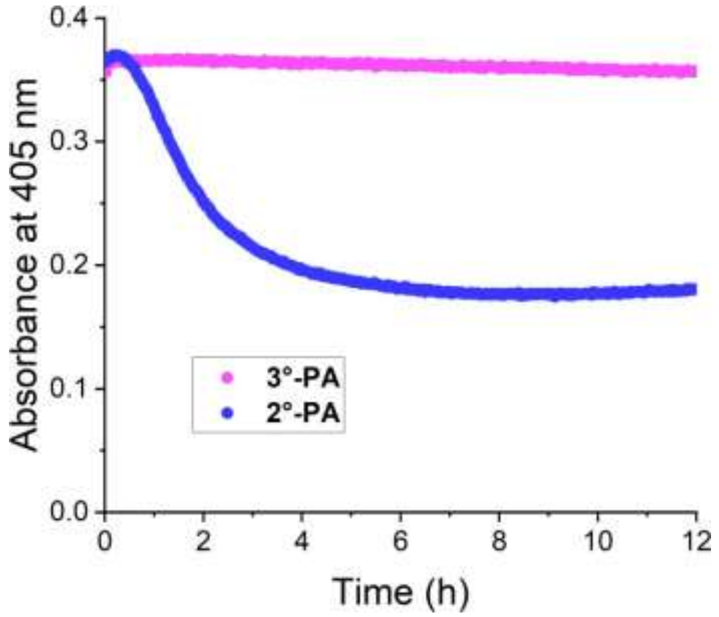

Figure S26. Hydrolytic stability test of **2°-PA** and **3°-PA**, displaying the absorbance at 405 nm over time (PBS-buffer/DMSO 99:1, 25 °C, 20 µM **2°-PA** or **3°-PA**).

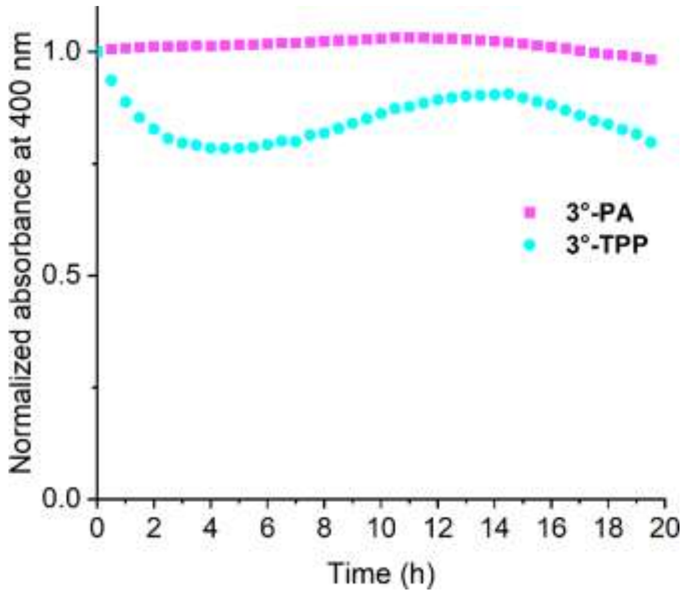

Figure S27. Hydrolytic stability test of **3°-PA** and **3°-TPP** at 37 °C, displaying the normalized absorbance at 400 nm over time (PBS-buffer/DMSO 99:1, 37 °C, 20 µM **3°-PA** or **3°-TPP**).

3.2 UPLC-MS

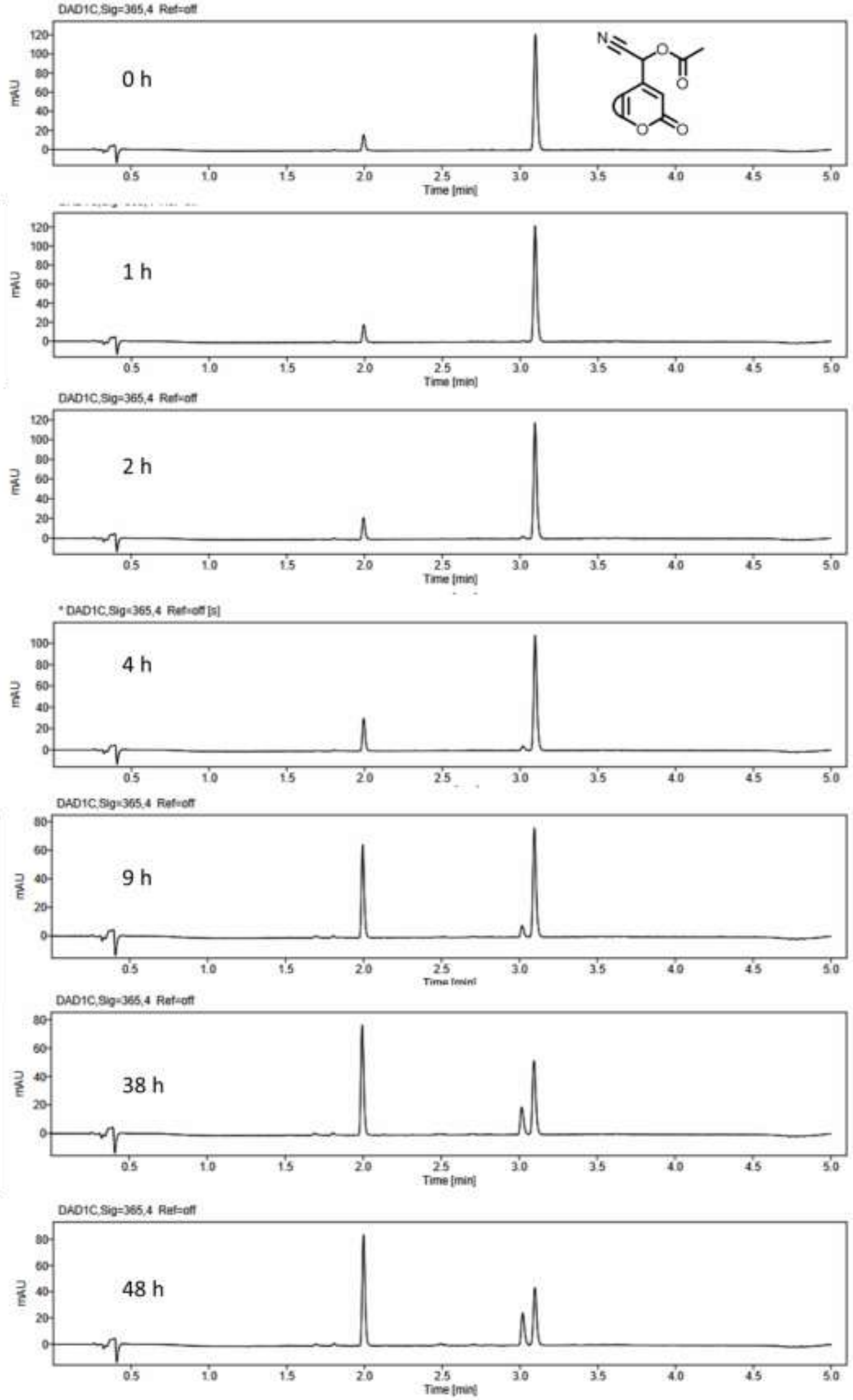

Figure S28. UPLC-MS chromatograms at λ = 365 nm of a sample of **2°-PA** (20 µM, water/DMSO 99:1) that was incubated at RT for the times indicated.

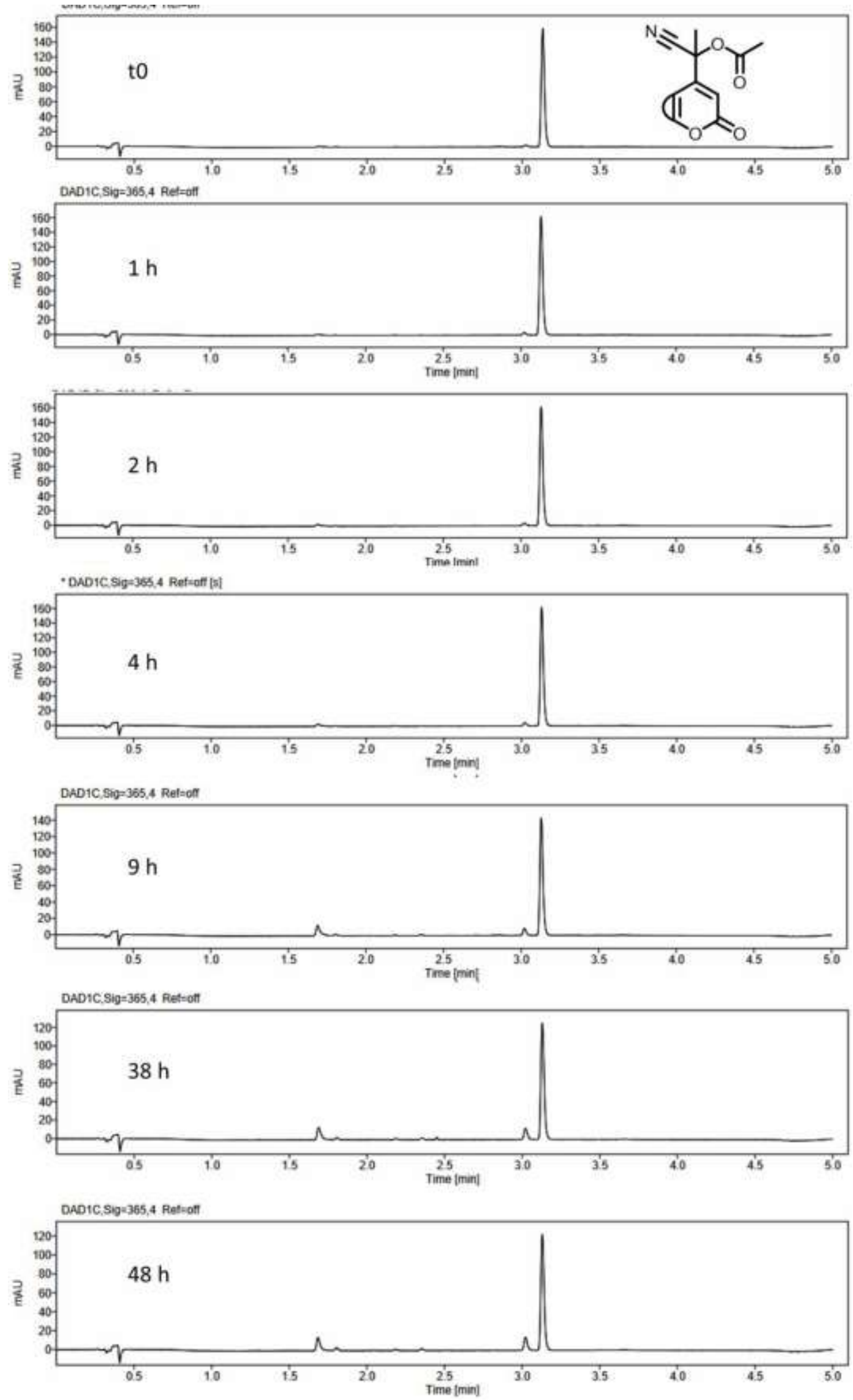

Figure S29. UPLC-MS chromatograms at λ = 365 nm of a sample of **3°-PA** (20 µM, water/DMSO 99:1) that was incubated at RT for the times indicated.

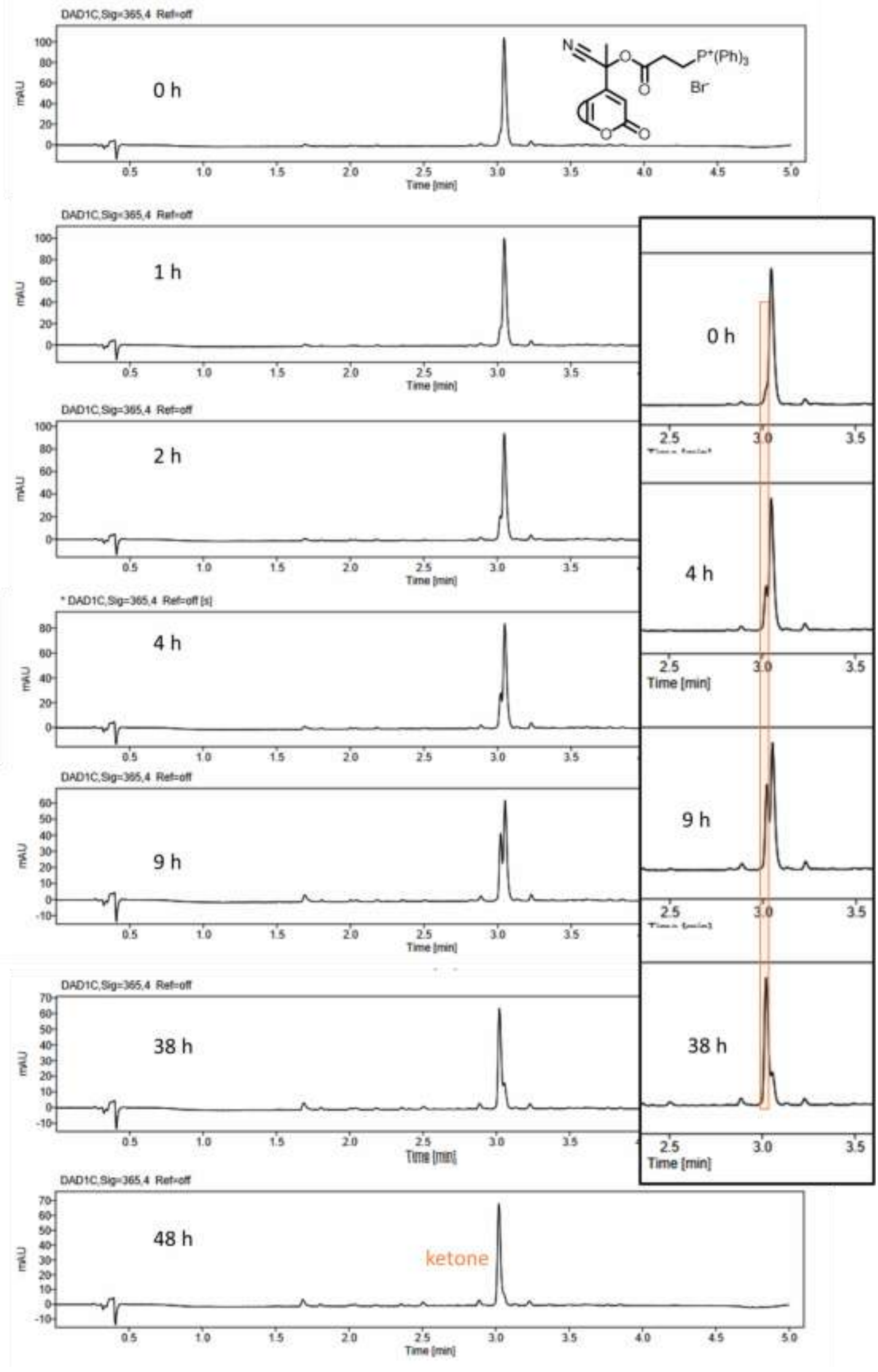

Figure S30. UPLC-MS chromatograms at λ = 365 nm of a sample of **3°-TPP** (20 µM, water/DMSO 99:1) that was incubated at RT for the times indicated. The insert shows the formation of the ketone (orange box) over time.

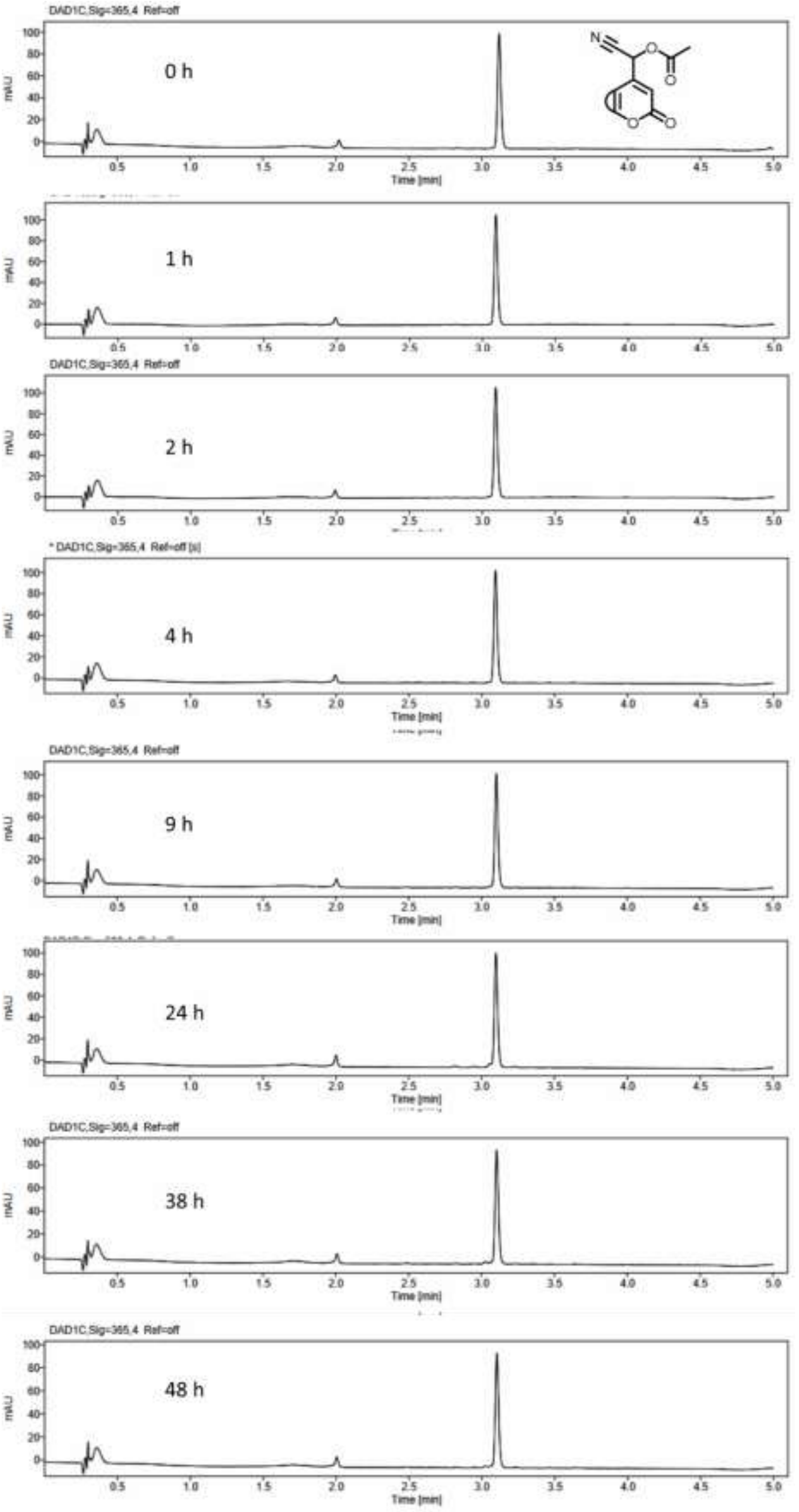

Figure S31. UPLC-MS chromatograms at λ = 365 nm of a sample of **2°-PA** (20 µM, MeCN/PBS-buffer 2:1) that was incubated at RT for the times indicated.

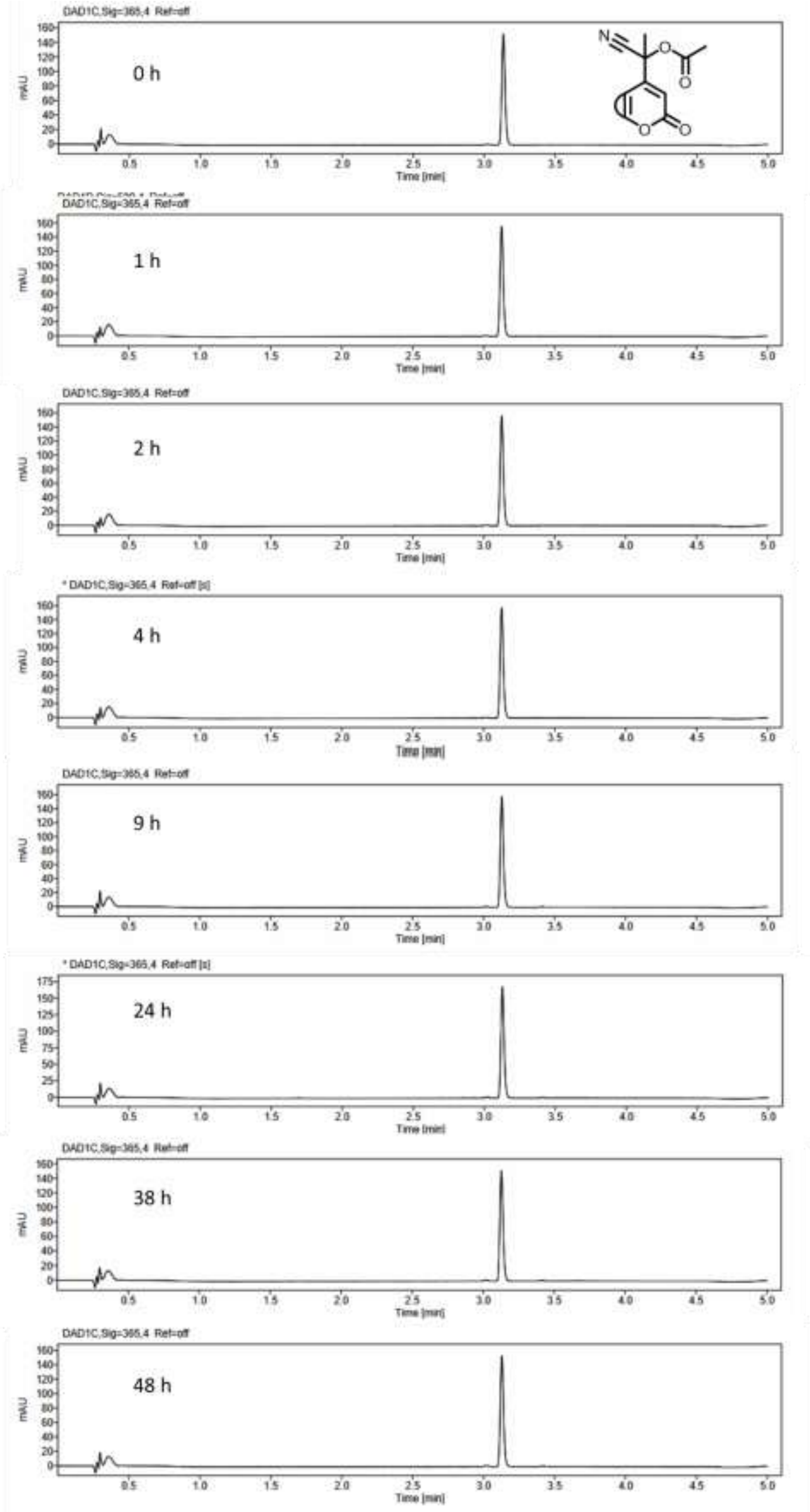

Figure S32. UPLC-MS chromatograms at λ = 365 nm of a sample of **3°-PA** (20 µM, MeCN/PBS-buffer 2:1) that was incubated at RT for the times indicated.

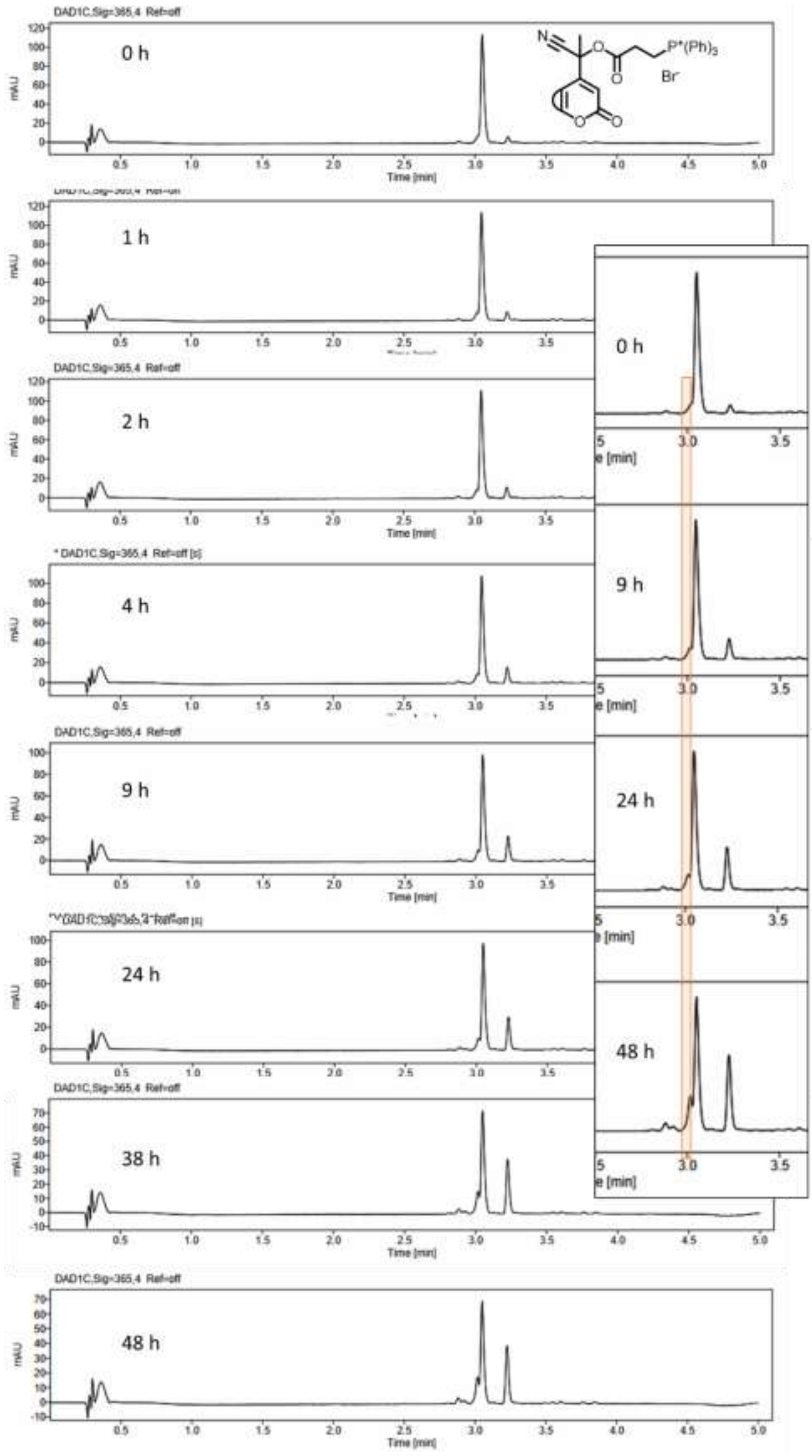

Figure S33. UPLC-MS chromatograms at λ = 365 nm of a sample of **3°-TPP** (20 µM, MeCN/PBS-buffer 2:1) that was incubated at RT for the times indicated. The insert shows the formation of the ketone (orange box) over time.

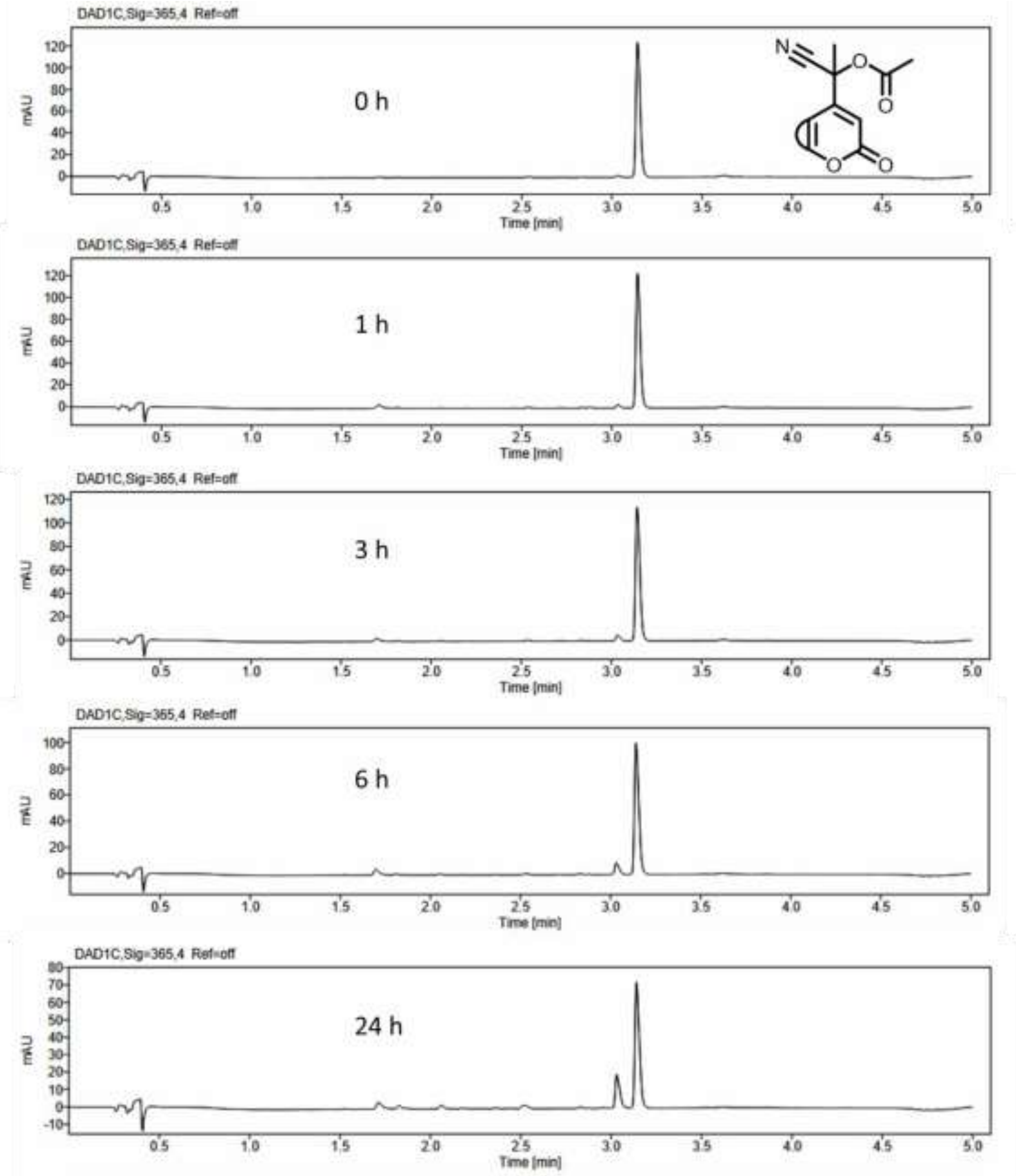

Figure S34. UPLC-MS chromatograms at λ = 365 nm of a sample of **3°-PA** (20 µM, DMSO/Phosphate-buffer -20 mM, pH 7.4-, 99:1) that was incubated at 37 °C for the times indicated.

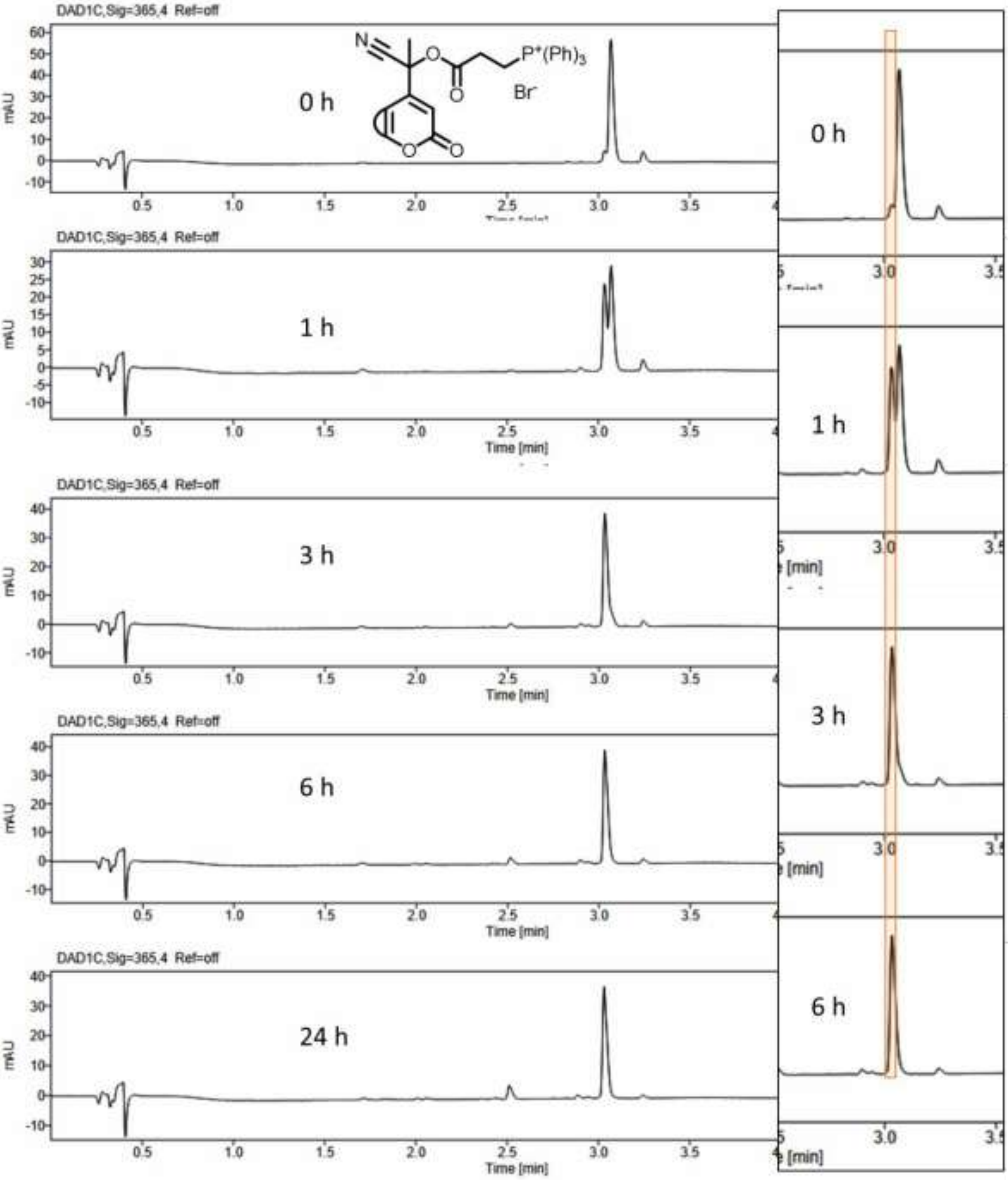

Figure S35. UPLC-MS chromatograms at λ = 365 nm of a sample of **3°-TPP** (20 µM, DMSO/Phosphate-buffer -20 mM, pH 7.4-, 99:1) that was incubated at 37 °C for the times indicated. The insert shows the formation of the ketone (orange box) over time.

4. Cell viability

4.1 Cell Culture

HEK239 cells (embryonic kidney carcinoma, human; ATCC® CRL-1™) were routinely cultured in DMEM supplemented with 10% FBS, 100 U mL−1 penicillin and 100 U mL−1 streptomycin. Cells were maintained at 37 °C in a humidified atmosphere containing 5% CO_2.

4.2 Cell viability assay

Cell viability was evaluated using the 3-(4,5-dimethylthiazol-2-yl)-2,5-diphenyl-2H-tetrazolium bromide (MTT) assay. In brief, cells were seeded at a concentration of 10,000 cells per well in a 96-well plate (150 µL). After 24 h, the corresponding amount of the compound to be tested (10 mM stock solutions in DMSO) was added, and the cells were incubated for 24h. Subsequently, irradiation was performed (λ = 400 nm), 96-well plates were irradiated at a fixed constant distance for 10, 5 or 1 min and then cultured at 37 °C for another 24 h. The supernatant was removed, and fresh medium (100 µL) was added together with MTT reagent (0.45 mg/mL final concentration) to each well. After 3 h incubation at 37 °C, the MTT solvent was added (100 µL/well) and absorbance was measured at 570 nm using a SpectraMax plate reader. The data were normalized to control, untreated cells, after background absorbance removal (measured at 750 nm).

**Dark**						
µM	**3°-PA**	SD	**3°-TPP**	SD	**4**	SD
**75**	77.68	9.17	74.32	8.09	96.85	11.33
**50**	82.38	5.43	83.89	8.42	100.56	6.45
**25**	89.81	11.38	88.96	10.64	95.44	10.57
**10**	98.67	8.06	93.88	11.67	84.83	3.52
**5**	97.50	12.69	92.72	15.07	93.08	10.25
**1**	114.83	23.20	98.28	17.92	92.98	16.34

**1 min 400 nm**						
µM	**3°-PA**	SD	**3°-TPP**	SD	**4**	SD
**75**	59.77	10.13	40.24	9.13	94.37	2.58
**50**	58.00	11.09	49.36	14.06	92.08	2.89
**25**	73.19	14.81	63.28	27.35	97.66	4.92
**10**	84.00	17.82	85.74	23.75	98.52	3.58
**5**	90.06	14.10	81.80	19.74	94.41	5.36
**1**	93.91	14.24	98.24	16.95	94.74	5.61

5 min 400 nm

µM	**3°-PA**	SD	**3°-TPP**	SD	**4**	SD
**75**	52.40	8.08	43.51	13.28	93.08	3.40
**50**	57.15	12.45	46.83	13.44	89.48	5.43
**25**	62.44	13.22	64.63	11.81	98.68	9.02
**10**	75.21	27.57	86.69	12.66	94.44	8.49
**5**	88.08	13.85	96.61	10.17	91.53	3.12
**1**	105.84	20.89	103.22	13.74	95.17	6.41

Table S1. Cell viability (%) of HEK239 cells after incubation with compounds at the concentration depicted in the left column, in the dark (top) or after irradiation for the times indicated (bottom two tables).

4.3 ANOVA-tests

Tukey's multiple comparisons test**. 5 minutes of irradiation.**

	Mean Diff.	95.00% CI of diff.	Below threshold?	Summary	Adjusted P Value
**75 µM**					
**3°-PA vs 3°-TPP**	8.900	-16.77 to 34.57	No	ns	0.6763
**3°-PA vs 4**	-40.68	-66.35 to -15.01	Yes	**	0.0012
**3°-TPP vs 4**	-49.58	-75.25 to -23.91	Yes	***	0.0001
**50 µM**					
**3°-PA vs 3°-TPP**	10.3	-15.37 to 35.97	No	ns	0.5935
**3°-PA vs 4**	-32.38	-58.05 to -6.711	Yes	*	0.0107
**3°-TPP vs 4**	-42.68	-68.35 to -17.01	Yes	***	0.0007
**25 µM**					
**3°-PA vs 3°-TPP**	-2.200	-27.87 to 23.47	No	ns	0.9761
**3°-PA vs 4**	-36.28	-61.95 to -10.61	Yes	**	0.004
**3°-TPP vs 4**	-34.08	-59.75 to -8.411	Yes	**	0.007
**10 µM**					
**3°-PA vs 3°-TPP**	-11.5	-37.17 to 14.17	No	ns	0.5233
**3°-PA vs 4**	-19.24	-44.91 to 6.429	No	ns	0.1737
**3°-TPP vs 4**	-7.740	-33.41 to 17.93	No	ns	0.7433
**5 µM**					
**3°-PA vs 3°-TPP**	-8.500	-34.17 to 17.17	No	ns	0.6997
**3°-PA vs 4**	-3.430	-29.10 to 22.24	No	ns	0.943
**3°-TPP vs 4**	5.070	-20.60 to 30.74	No	ns	0.8799
**1 µM**					
**3°-PA vs 3°-TPP**	2.600	-23.07 to 28.27	No	ns	0.9668
**3°-PA vs 4**	10.63	-15.04 to 36.30	No	ns	0.5741
**3°-TPP vs 4**	8.030	-17.64 to 33.70	No	ns	0.7268

Table S2. Results of the ANOVA test evaluating the statistical relevance of the difference in cytotoxic effect between the three tested compounds after 5 minutes of irradiation. A p value < 0.05 was considered significant.

Tukey's multiple comparisons test. **1 minute of irradiaton**

	Mean Diff.	95.00% CI of diff.	Below threshold?	Summary	Adjusted P Value
**75 µM**					
**3°-PA vs 3°-TPP**	19.6	-8.413 to 47.61	No	ns	0.2153
**3°-PA vs 4**	-34.6	-62.61 to -6.587	Yes	*	0.0126
**3°-TPP vs 4**	-54.2	-82.21 to -26.19	Yes	****	<0.0001
**50 µM**					
**3°-PA vs 3°-TPP**	8.600	-19.41 to 36.61	No	ns	0.7353
**3°-PA vs 4**	-34.1	-62.11 to -6.087	Yes	*	0.0141
**3°-TPP vs 4**	-42.7	-70.71 to -14.69	Yes	**	0.0019
**25 µM**					
**3°-PA vs 3°-TPP**	9.900	-18.11 to 37.91	No	ns	0.6662
**3°-PA vs 4**	-24.5	-52.51 to 3.513	No	ns	0.0964
**3°-TPP vs 4**	-34.4	-62.41 to -6.387	Yes	*	0.0132
**10 µM**					
**3°-PA vs 3°-TPP**	-1.700	-29.71 to 26.31	No	ns	0.9879
**3°-PA vs 4**	-14.5	-42.51 to 13.51	No	ns	0.4237
**3°-TPP vs 4**	-12.8	-40.81 to 15.21	No	ns	0.5101
**5 µM**					
**3°-PA vs 3°-TPP**	8.300	-19.71 to 36.31	No	ns	0.7509
**3°-PA vs 4**	-4.300	-32.31 to 23.71	No	ns	0.9255
**3°-TPP vs 4**	-12.6	-40.61 to 15.41	No	ns	0.5206
**1 µM**					
**3°-PA vs 3°-TPP**	-4.300	-32.31 to 23.71	No	ns	0.9255
**3°-PA vs 4**	-0.8	-28.81 to 27.21	No	ns	0.9973
**3°-TPP vs 4**	3.500	-24.51 to 31.51	No	ns	0.95

Table S3. Results of the ANOVA test evaluating the statistical relevance of the difference in cytotoxic effect between the three tested compounds after 1 minute of irradiation. A p value < 0.05 was considered significant.

Tukey's multiple comparisons test. **Irradiation time – 3°-TPP**					
	Mean Diff.	95.00% CI of diff.	Below threshold?	Summary	Adjusted P Value
**5 vs 1 min**	3.300	-27.01 to 33.61	No	ns	0.9618
**5 vs 0 min**	-30.8	-61.11 to -0.4852	Yes	*	0.0458
**1 vs 0 min**	-34.1	-64.41 to -3.785	Yes	*	0.0246
**50**					
**5 vs 1 min**	-2.600	-32.91 to 27.71	No	ns	0.9761
**5 vs 0 min**	-37.1	-67.41 to -6.785	Yes	*	0.0135
**1 vs 0 min**	-34.5	-64.81 to -4.185	Yes	*	0.0227
**25**					
**5 vs 1 min**	1.300	-29.01 to 31.61	No	ns	0.994
**5 vs 0 min**	-24.4	-54.71 to 5.915	No	ns	0.135
**1 vs 0 min**	-25.7	-56.01 to 4.615	No	ns	0.11
**10**					
**5 vs 1 min**	1.000	-29.31 to 31.31	No	ns	0.9964
**5 vs 0 min**	-7.200	-37.51 to 23.11	No	ns	0.8313
**1 vs 0 min**	-8.200	-38.51 to 22.11	No	ns	0.7873
**5**					
**5 vs 1 min**	14.8	-15.51 to 45.11	No	ns	0.4648
**5 vs 0 min**	3.900	-26.41 to 34.21	No	ns	0.947
**1 vs 0 min**	-10.9	-41.21 to 19.41	No	ns	0.6569
**1**					
**5 vs 1 min**	5.000	-25.31 to 35.31	No	ns	0.9145
**5 vs 0 min**	4.900	-25.41 to 35.21	No	ns	0.9178
**1 vs 0 min**	-0.1	-30.41 to 30.21	No	ns	>0.9999

Table S4. Results of the ANOVA test evaluating the statistical relevance of the difference in cytotoxic effect between the different irradiation times for compound **3°-TPP**. A p value < 0.05 was considered significant.

5. Computational data

5.1 Overview of Methods and Results

All computational input files were prepared in GaussView 6.0 on a local Windows 10 terminal. Input files were then transferred to the Rijksuniversiteit Groningen Peregrine HPC cluster where DFT or TD-DFT calculations were carried out using the Gaussian 16 (g16) suite of programs.

The DFT thermochemistry of heterolysis for coumarin PPGs **2°-PA** and **3°-PA** was examined. Geometry optimization of structures to either excited state S_1 or T_1 minima or transition states (TS) were done using the g16 *opt* command at the MN15 functional and Def2SVP basis set level of theory with implicit solvation using the Solvation Model based on Density (SMD = water).[8–10] Transition state geometry inputs were the result of rational guess based on bond-breaking atomic distances, or were the result of potential energy surface relaxed coordinate scans using the g16 *scan* command at the MN15/Def2SVP/SMD=water level. For both **2°-PA** and **3°-PA**, S_1 or T_1 heterolysis of both acetoxy anion or cyanide anion payloads were considered. S_1 and T_1 acetoxy anion heterolysis events for both **2°-PA** and **3°-PA** were found to be kinetically facile (+5.9 to +8.3 kcal/mol). In the case of cyanide anion heterolysis, the S_1 cyanide payload bond breaking event for **2°-PA** was found to be kinetically prohibitive (+23.5 kcal/mol), whereas no cyanide heterolysis bond breaking event was found on the S_1 potential energy surface calculated for **3°-PA**. Additionally, no T_1 cyanide heterolysis bond breaking events were found for either **2°-PA** and **3°-PA**. Intrinsic reaction coordinate (IRC)iv calculations were carried out on the transition state structures to verify that they connected to the associated reactant and product minima structures.

The S_1 optimizations of solvent-encapsulated contact ion pair (CIP) structures arising from the acetate heterolysis of **2°-PA** and **3°-PA** were both unsuccessful, despite favorable S_1 TS kinetics for both. It is unclear if a competing S_1 photochemical process dominates or whether this is due to instability of the insipient CIP. The T_1 solvent-encapsulated contact ion pair (CIP) structures arising from the triplet state acetate heterolysis of **2°-PA** and **3°-PA** yielded interesting insights. The incipient positive charge left on the PPG after acetate heterolysis is highly delocalized, with the nitrile nitrogen taking a partial δ^+ nature. Finally, no heterolysis transition states were found for **2°-PA** and **3°-PA** on the S_0 potential energy surface, suggesting that the aqueous labile nature of **2°-PA** may arise from a different decomposition or solvolysis pathway.

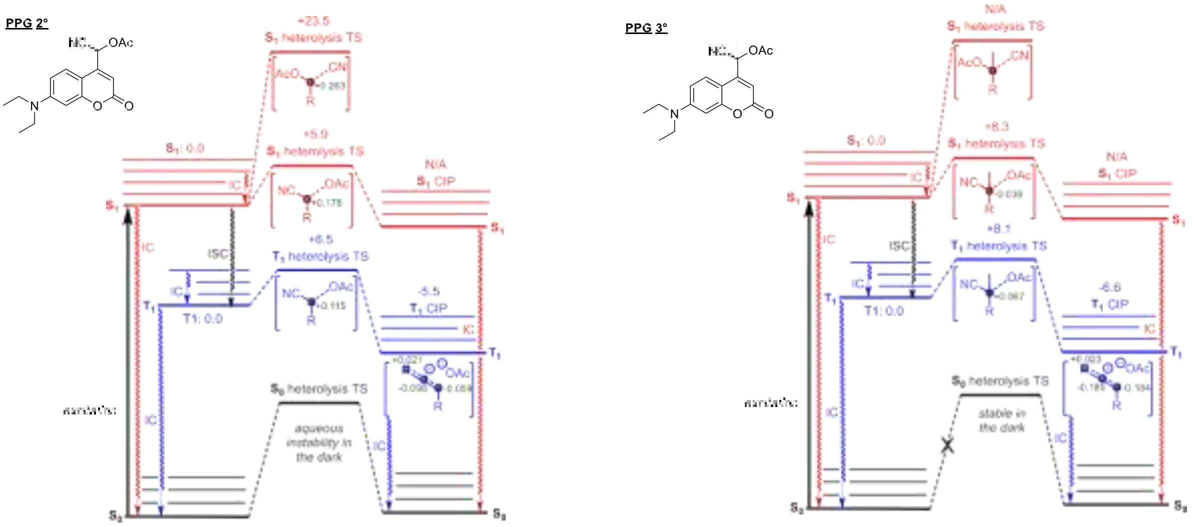

Figure S36. The S_1 and T_1 excited state barriers and thermochemistry for the uncaging processes for **2°-PA** and **3°-PA** as calculated by DFT. Shown are the ZPE-corrected free energies of structures optimized at the MN15/Def2SVP/SMD=H2O level (except for **Y** S_1 excited state CIP, where the single point energy is shown), and all values are in kcal/mol.

After optimization, frequency DFT calculations of the optimized structures were carried out using the g16 *freq* command at the MN15/Def2SVP/SMD=water level, to confirm that minima structures had zero imaginary frequencies and that transition states had a single imaginary frequency. All shown free energies (Figure S36) are zero point energy (ZPE) and thermally corrected and were obtained from the frequency calculations. All shown free energies are reported in kcal/mol, at 298.15 K and 1 atm.

5.2. Optimized Geometries and XYZ Coordinates

PPG 2°-PA (S_1) optimized geometry [# opt=calcfc freq scrf=(smd,solvent=water) def2svp mn15]

EE + Thermal Free Energy Correction: -1066.543882 Ha

0 1

C	1.89477400	0.79384000	-0.22424000
C	2.24303000	2.10644400	0.16751200
C	1.28440400	3.10477600	0.46232400
O	-0.06409800	2.74116400	0.37430700
C	-0.45231600	1.47273900	0.05047400
C	0.50771200	0.45449300	-0.25439900
C	-0.01802400	-0.83995700	-0.55291400
C	-1.36973600	-1.09451100	-0.58849700
C	-2.32106400	-0.05989800	-0.30736700
N	-3.66031200	-0.28291000	-0.34412900
C	-4.18285600	-1.62370600	-0.58810100
C	-4.57552100	0.78054900	0.09285900
C	-1.80519800	1.23323800	0.02866400
H	3.28787000	2.41667000	0.24148800
H	0.66884800	-1.66179100	-0.76620000
H	-1.69984500	-2.10505900	-0.82255000
H	-5.19646700	-1.52411500	-0.99144100
H	-3.58931400	-2.09684300	-1.38092700
H	-4.32445300	1.03969900	1.13636300
H	-4.35966700	1.67965800	-0.50529600
H	-2.45926700	2.06855900	0.27110800
C	2.90764300	-0.25959500	-0.52638500
O	3.05989400	-1.14837700	0.60297400
C	3.07996600	-2.47932600	0.36848100
O	3.01060400	-2.94539100	-0.74880400
C	3.17985400	-3.25465700	1.63895900
H	3.21977600	-4.32629100	1.41929800

H	4.07727800	-2.94017200	2.18931500
H	2.30787400	-3.02538400	2.26771100
C	-4.19604200	-2.48136800	0.67101800
H	-4.60070400	-3.47637700	0.43803300
H	-3.17959200	-2.60413000	1.07425800
H	-4.82510100	-2.02442400	1.44940800
C	-6.05181400	0.46185900	-0.01302100
H	-6.36285200	0.27609200	-1.05080500
H	-6.34317800	-0.39788000	0.60676100
H	-6.60832900	1.33716800	0.34918900
O	1.48919400	4.27737500	0.78884700
H	2.60256900	-0.87013200	-1.39288300
C	4.22910800	0.29975800	-0.84668200
N	5.27406600	0.73269800	-1.10494600

PPG 2°-PA (S_1 OAc TS) optimized geometry [# opt=(calcfc,ts,noeigentest) freq td=(root=1) scrf=(smd,solvent=water) def2svp mn15]

EE + Thermal Free Energy Correction: -1066.534407 Ha

0 1

C	1.94138700	0.83226900	-0.33279500
C	2.29267700	2.10045500	0.14544800
C	1.33628400	3.09553100	0.50585800
O	-0.00589500	2.75713400	0.40105500
C	-0.40757700	1.51216800	0.02788600
C	0.53059300	0.50759200	-0.34244200
C	0.00846500	-0.77337800	-0.67084100
C	-1.33810800	-1.03088400	-0.67837500
C	-2.28310400	-0.00046200	-0.33542600
N	-3.61192000	-0.22943000	-0.33896900
C	-4.14283100	-1.56503400	-0.61163900
C	-4.52549700	0.82351900	0.13908100
C	-1.76360700	1.27916500	0.03221000
H	3.33906500	2.40250400	0.22950900
H	0.69524900	-1.58444700	-0.92014500
H	-1.67619700	-2.03256600	-0.93517100
H	-5.16267300	-1.44893800	-0.99274600
H	-3.56576700	-2.01692100	-1.42696100
H	-4.25037400	1.05622500	1.18115500
H	-4.32074100	1.73190700	-0.44708500
H	-2.40948700	2.10321900	0.32750200
C	2.89939000	-0.16931200	-0.70664700
O	3.06779300	-1.27415900	0.69491900
C	2.87969500	-2.54154800	0.43948900
O	2.66717500	-3.00513100	-0.68104900
C	2.92514600	-3.40670100	1.67309500
H	3.84271500	-3.20234200	2.24103900

H	2.07228300	-3.14938000	2.31786400
H	2.87349400	-4.46578100	1.39581800
C	-4.13197300	-2.44834100	0.62812700
H	-4.55318900	-3.43299600	0.38241800
H	-3.10635900	-2.58999800	1.00066000
H	-4.73553900	-2.00300400	1.43271100
C	-6.00185600	0.50117400	0.05645700
H	-6.33539800	0.34085100	-0.97839200
H	-6.27803000	-0.37301800	0.66252700
H	-6.55072100	1.36675500	0.45195000
O	1.57281600	4.23369900	0.89332700
H	2.59546900	-0.92836300	-1.43272500
C	4.25954400	0.26053800	-0.87790900
N	5.36039400	0.61135000	-1.01894600

PPG 2°-PA (S_1 CN TS) optimized geometry [# opt=(calcfc,ts,noeigentest) freq td=(root=1) scrf=(smd,solvent=water) def2svp mn15]

EE + Thermal Free Energy Correction: -1066.506446 Ha

0 1

C	-1.70986700	0.51127700	-0.20613800
C	-2.08804100	1.84329600	-0.12620900
C	-1.14089500	2.92103200	-0.03319000
O	0.19550300	2.58691400	-0.00435900
C	0.62329600	1.29640400	-0.02421200
C	-0.29212100	0.21409000	-0.11944200
C	0.25055000	-1.09984000	-0.10937200
C	1.59835700	-1.32536400	-0.02853000
C	2.52231300	-0.22275000	0.05966200
N	3.85029500	-0.42002000	0.14380000
C	4.41601100	-1.76938300	0.08837700
C	4.75628400	0.74478300	0.12398200
C	1.98214800	1.10058400	0.05485700
H	-3.13827500	2.13458800	-0.15713600
H	-0.42346300	-1.95427700	-0.16282700
H	1.95872300	-2.35171300	-0.03649400
H	5.38310300	-1.74626900	0.60091300
H	3.77936900	-2.44347900	0.67372300
H	4.55548500	1.30460200	-0.80343200
H	4.46855400	1.40342200	0.95739500
H	2.61188500	1.98481500	0.12061800
C	-2.65506300	-0.54222400	-0.28685700
O	-3.95753300	-0.17880800	-0.40821500
C	-4.89689000	-1.16467700	-0.48040100
O	-4.58883000	-2.33268900	-0.51287700
C	-6.27316000	-0.59811200	-0.50725600
H	-7.00310200	-1.40010400	-0.65493200

H	-6.46474000	-0.08376600	0.44580100
H	-6.35031000	0.14955700	-1.30853500
C	4.57811600	-2.26665300	-1.34109100
H	5.01957500	-3.27270600	-1.33088600
H	3.60511000	-2.31747400	-1.85226900
H	5.23901700	-1.60270800	-1.91710500
C	6.23352800	0.43200100	0.22433000
H	6.49569900	-0.04299900	1.17974500
H	6.58789900	-0.20082200	-0.60133000
H	6.77538200	1.38603400	0.16859200
O	-1.40913200	4.10917100	0.01379200
H	-2.39740700	-1.53744900	-0.64220900
C	-2.57162200	-1.35735400	1.80574100
N	-1.65318500	-1.74978200	2.43450700

PPG 2°-PA (T_1) optimized geometry [# opt=calcfc freq scrf=(smd,solvent=water) def2svp mn15]

EE + Thermal Free Energy Correction: -1066.573284 Ha

0 3

C	1.88590900	0.77796700	-0.24255100
C	2.24118400	2.09225600	0.18905900
C	1.28209300	3.08559100	0.49519600
O	-0.05978700	2.73183100	0.38414300
C	-0.45494300	1.46436500	0.04301500
C	0.50817400	0.44902400	-0.27816500
C	-0.02485300	-0.83666400	-0.59429600
C	-1.37917500	-1.08530800	-0.62759200
C	-2.32523000	-0.05842400	-0.32705900
N	-3.66867400	-0.28348000	-0.35294200
C	-4.19217700	-1.62878800	-0.58076300
C	-4.58082900	0.77691900	0.10395700
C	-1.80510600	1.22900900	0.02173900
H	3.28471100	2.39715600	0.28048400
H	0.65794800	-1.65904300	-0.81664200
H	-1.71172400	-2.09163300	-0.87674300
H	-5.20849600	-1.53268900	-0.97763800
H	-3.60449400	-2.10829100	-1.37288700
H	-4.32395800	1.01633300	1.14958000
H	-4.36230900	1.68094700	-0.48367100
H	-2.45591200	2.06299000	0.27844300
C	2.90515200	-0.26611500	-0.54462600
O	3.11818600	-1.13443500	0.59654800
C	3.09530200	-2.46908800	0.40006400
O	2.94847900	-2.96873800	-0.69598500
C	3.25378900	-3.21203600	1.68474900
H	3.28242100	-4.28917100	1.49158800
H	4.17407900	-2.88472100	2.18749200

H	2.40996800	-2.96631600	2.34515300
C	-4.19191100	-2.46840400	0.68880200
H	-4.60822400	-3.46298900	0.47631600
H	-3.16996600	-2.59408700	1.07693800
H	-4.80444800	-1.99626600	1.47142300
C	-6.05786300	0.46260200	-0.00387000
H	-6.36903900	0.28444000	-1.04293900
H	-6.35167700	-0.40025000	0.61034600
H	-6.61199300	1.33694100	0.36419900
O	1.49873200	4.24452800	0.84916100
H	2.59133300	-0.90328900	-1.38730200
C	4.20595300	0.31165300	-0.91327800
N	5.23211600	0.76592400	-1.20868200

PPG 2°-PA (T_1 OAc TS) optimized geometry [# opt=(calcfc,ts,noeigentest) freq scrf=(smd,solvent=water) def2svp mn15]

EE + Thermal Free Energy Correction: -1066.562899 Ha

0 3

C	1.77070700	0.89110500	-0.30899200
C	2.06153300	2.19032000	0.17718300
C	1.05398900	3.14014800	0.50844200
O	-0.26551700	2.73888200	0.37666600
C	-0.60776500	1.46880600	0.01953300
C	0.38182500	0.50350600	-0.32540500
C	-0.07656200	-0.80244500	-0.63531800
C	-1.41232000	-1.12293600	-0.64939800
C	-2.40360600	-0.13476800	-0.33225200
N	-3.72350300	-0.43094800	-0.33567100
C	-4.18708900	-1.80038800	-0.56778700
C	-4.69276200	0.58949300	0.10565500
C	-1.95059100	1.17399100	0.01388100
H	3.09013300	2.53865600	0.28003500
H	0.65031100	-1.58475100	-0.85918800
H	-1.70180800	-2.14339300	-0.89148700
H	-5.20848800	-1.74481500	-0.95785700
H	-3.58200900	-2.25040800	-1.36295800
H	-4.43485200	0.86264000	1.14181000
H	-4.52408900	1.48768900	-0.50634300
H	-2.63739600	1.97198500	0.28847400
C	2.77659000	-0.05133900	-0.66995100
O	3.01539400	-1.16335600	0.73374100
C	3.58828800	-2.28990200	0.40863800
O	4.06524000	-2.52250300	-0.70248800
C	3.60778200	-3.30258500	1.52601900
H	4.18312800	-4.18668200	1.22800900

H	4.04119200	-2.85241300	2.42961500
H	2.57492500	-3.59381400	1.76585700
C	-4.14096200	-2.63571500	0.70360600
H	-4.51881200	-3.64542600	0.49210200
H	-3.11111900	-2.71926300	1.08167800
H	-4.76571200	-2.18826100	1.49062300
C	-6.15053900	0.19266600	0.02198000
H	-6.46586700	-0.01691800	-1.00967800
H	-6.38971500	-0.67363200	0.65443500
H	-6.74374700	1.04317700	0.38472900
O	1.23264700	4.28855900	0.89288100
H	2.51816200	-0.83695200	-1.38445500
C	4.11352900	0.44865000	-0.86164500
N	5.18798500	0.86641800	-1.01817600

PPG 2°-PA (T_1 CIP) optimized geometry [# opt=(calcfc,noeigentest) freq scrf=(smd,solvent=water) def2svp mn15]

EE + Thermal Free Energy Correction: -1066.582106 Ha

0 3

C	1.91960200	1.13907600	-0.28462100
C	2.17746600	2.45790100	0.08794100
C	1.12422600	3.39852500	0.41830000
O	-0.15914900	2.93471200	0.36071500
C	-0.47362100	1.65347300	0.01944800
C	0.53348800	0.70926800	-0.31587600
C	0.12223900	-0.60340100	-0.65598500
C	-1.20389800	-0.95346600	-0.66817600
C	-2.22012400	0.00556000	-0.32582700
N	-3.52574700	-0.33151700	-0.31367900
C	-3.95456500	-1.70792500	-0.58133900
C	-4.52748900	0.65416400	0.14239800
C	-1.80954400	1.32821900	0.02113500
H	3.19457000	2.84845400	0.13949400
H	0.87442400	-1.35998800	-0.89599700
H	-1.47109900	-1.97462900	-0.93114500
H	-4.98181500	-1.66540000	-0.95640900
H	-3.34538900	-2.11614700	-1.39510000
H	-4.25074900	0.94210400	1.16885100
H	-4.40483800	1.55149400	-0.48173300
H	-2.51718700	2.10778100	0.29474000
C	2.96949400	0.24888600	-0.61950000
O	2.34361400	-2.09880900	1.22272500
C	2.71424100	-2.94440300	0.36944000
O	2.51797200	-2.85924300	-0.87959600
C	3.50517700	-4.14985200	0.86220100
H	3.39873900	-5.00152900	0.17758100

H	4.57150600	-3.87564400	0.89941600
H	3.19760800	-4.43300700	1.87778400
C	-3.86978900	-2.57567100	0.66688900
H	-4.22758200	-3.58594700	0.42644200
H	-2.83278800	-2.64665200	1.02683400
H	-4.49415000	-2.16754400	1.47480400
C	-5.96944600	0.20016500	0.09942200
H	-6.30879800	-0.01452600	-0.92329300
H	-6.15569800	-0.67696300	0.73422800
H	-6.58119500	1.02672900	0.48572900
O	1.29971500	4.55501200	0.73642200
H	2.78030600	-0.79098500	-0.91897300
C	4.31757600	0.65402100	-0.55743100
N	5.44099100	0.97320100	-0.51430700

PPG 3°-PA (S_1) optimized geometry [# opt=calcfc freq td=(root=1) scrf=(smd,solvent=water) def2svp mn15]

EE + Thermal Free Energy Correction: -1105.739597 Ha

0 1

C	1.65917200	0.85748900	-0.09169400
C	1.92562100	2.21697800	0.19873900
C	0.91951600	3.18537700	0.41564600
O	-0.40605300	2.75311400	0.33709400
C	-0.72386700	1.45088400	0.07220000
C	0.28760500	0.45361500	-0.14571900
C	-0.20347600	-0.87161300	-0.38996200
C	-1.54552700	-1.17044800	-0.44449600
C	-2.53782300	-0.15790000	-0.24827100
N	-3.86894700	-0.42463300	-0.30831200
C	-4.34276200	-1.79730500	-0.45171500
C	-4.82769500	0.63513200	0.03154200
C	-2.06723700	1.16562000	0.02569700
H	2.94783400	2.59213500	0.26701100
H	0.50934000	-1.68265200	-0.52821700
H	-1.83566900	-2.20325200	-0.62981700
H	-5.34639400	-1.76641100	-0.88975200
H	-3.71121000	-2.31764000	-1.18322100
H	-4.62982100	0.95809300	1.06916500
H	-4.60948600	1.50581700	-0.60600800
H	-2.75039400	1.99370900	0.20457500
C	2.77341500	-0.11042800	-0.43525600
O	2.59299700	-1.26878200	0.40458000
C	3.50922600	-2.25765100	0.41495000
O	4.55767300	-2.17882200	-0.18813000
C	3.05452900	-3.41328400	1.24222400
H	3.87048100	-4.13469100	1.35340700

H	2.70955700	-3.05917100	2.22258900
H	2.19959200	-3.89081400	0.74074000
C	-4.36614300	-2.54196500	0.87732200
H	-4.73867400	-3.56481500	0.72531400
H	-3.35663000	-2.59930700	1.31177600
H	-5.02584200	-2.03540900	1.59773100
C	-6.28907700	0.26956600	-0.12072700
H	-6.54283500	0.00598200	-1.15730000
H	-6.58886600	-0.55538000	0.54095500
H	-6.88460600	1.15077300	0.15479400
O	1.06702000	4.38553800	0.66843600
C	4.08817800	0.50233900	-0.12246100
N	5.10660300	1.01525200	0.09076400
C	2.75303800	-0.48078600	-1.92503000
H	2.91088000	0.43364000	-2.51310600
H	3.54180800	-1.20424600	-2.16459500
H	1.77410100	-0.90279100	-2.18697900

PPG 3°-PA (S_1 OAc TS) optimized geometry [# opt=(calcfc,ts,noeigentest) freq td=(root=1) scrf=(smd,solvent=water) def2svp mn15]

EE + Thermal Free Energy Correction: -1105.726317 Ha

0 1

C	-1.62203200	0.91396200	0.23006200
C	-1.86891800	2.17870600	-0.32645800
C	-0.84514400	3.13079500	-0.60412100
O	0.46256800	2.75650500	-0.32471100
C	0.77800800	1.46700400	-0.03080000
C	-0.23259100	0.49314400	0.22171300
C	0.21214800	-0.85383300	0.35927500
C	1.54049600	-1.18988600	0.38321800
C	2.55471300	-0.17899500	0.24122300
N	3.86731500	-0.47636200	0.30162300
C	4.31873400	-1.86156800	0.43532100
C	4.85929600	0.57557900	0.01622800
C	2.11838300	1.15911700	-0.00597100
H	-2.88691400	2.53406200	-0.49755400
H	-0.53261300	-1.64526300	0.42774400
H	1.81179200	-2.23798900	0.49088900
H	5.30522200	-1.84790500	0.91062100
H	3.65445800	-2.38524300	1.13288400
H	4.68827600	0.92669300	-1.01515000
H	4.63933400	1.42719500	0.67648000
H	2.81774900	1.96961000	-0.19936600
C	-2.67814200	0.04125500	0.70490800
O	-2.70421500	-1.27155100	-0.55095500
C	-3.68731100	-2.12977800	-0.55965100
O	-4.63207000	-2.11662500	0.22785400
C	-3.55106000	-3.17985800	-1.63465400
H	-4.48631100	-3.74331500	-1.73355000

H	-3.27807000	-2.71340400	-2.59033600
H	-2.73952000	-3.86850400	-1.35637000
C	4.38141700	-2.57297500	-0.90884300
H	4.74531700	-3.59994300	-0.76654000
H	3.38604600	-2.61627200	-1.37604600
H	5.06560500	-2.05391300	-1.59635100
C	6.30823900	0.17508800	0.19216400
H	6.53463600	-0.11460900	1.22789300
H	6.60677100	-0.63989400	-0.48190900
H	6.92504100	1.05102800	-0.05112500
O	-0.99776500	4.27124300	-1.02791100
C	-3.99750400	0.62457800	0.56146200
N	-5.04172100	1.12980800	0.47362100
C	-2.52015300	-0.70801700	2.01184200
H	-3.19863300	-1.56890200	2.04593300
H	-1.49037800	-1.04205400	2.17381400
H	-2.78659100	-0.02800000	2.83562300

PPG 3°-PA (T_1) optimized geometry [# opt=calcfc freq scrf=(smd,solvent=water) def2svp mn15]

EE + Thermal Free Energy Correction: -1105.726317 Ha

0 3

C	1.65108900	0.85210000	-0.10520700
C	1.92088600	2.22417200	0.18988800
C	0.91152700	3.18609900	0.41831100
O	-0.40581400	2.75306100	0.34838700
C	-0.72739000	1.44858900	0.06986600
C	0.29099500	0.45562900	-0.16099200
C	-0.20328300	-0.86542500	-0.41710000
C	-1.54739700	-1.16226600	-0.46849700
C	-2.53782600	-0.15775300	-0.25976500
N	-3.87341600	-0.42823700	-0.31332000
C	-4.34546100	-1.80432100	-0.44618600
C	-4.83269600	0.62793400	0.04370300
C	-2.06687700	1.16372600	0.02303200
H	2.94033000	2.60323200	0.25517400
H	0.50735700	-1.67623800	-0.56528300
H	-1.83612900	-2.19402800	-0.66311000
H	-5.34914900	-1.77702300	-0.88406700
H	-3.71401200	-2.32798000	-1.17413000
H	-4.63267600	0.93198000	1.08532300
H	-4.61254300	1.50470400	-0.58287500
H	-2.74960800	1.99070300	0.21036400
C	2.76911700	-0.11424900	-0.44254900
O	2.59308900	-1.27401500	0.40097400
C	3.51827200	-2.25233600	0.42589500
O	4.57300800	-2.16758200	-0.16639100
C	3.06774400	-3.40888700	1.25490300
H	3.88981700	-4.12155700	1.37697400
H	2.71064200	-3.05361400	2.23050400

H	2.22210400	-3.89759200	0.74855100
C	-4.36446400	-2.53378300	0.88964300
H	-4.74555900	-3.55527100	0.75166500
H	-3.35228900	-2.59409000	1.31734300
H	-5.01510200	-2.01515800	1.60983800
C	-6.29375800	0.26405200	-0.11579400
H	-6.54422600	0.00911100	-1.15532800
H	-6.59611700	-0.56604400	0.53818100
H	-6.88976600	1.14318000	0.16518200
O	1.06931200	4.38049000	0.67527200
C	4.07940700	0.50556700	-0.12386100
N	5.09112700	1.02983000	0.09440000
C	2.76547600	-0.49832800	-1.92881300
H	3.55121300	-1.22978000	-2.15435600
H	1.78598400	-0.91387100	-2.19892800
H	2.93682900	0.40943600	-2.52338500

PPG 3°-PA (T_1 TS) optimized geometry [# opt=(calcfc,ts,noeigentest) freq=noraman scrf=(smd,solvent=water) def2svp mn15]

EE + Thermal Free Energy Correction: -1105.755085 Ha

0 3

C	1.59476400	0.88675000	-0.25208200
C	1.85497800	2.17255900	0.29449600
C	0.83631700	3.12326000	0.57616200
O	-0.47363200	2.73833800	0.33752600
C	-0.79621700	1.45061400	0.03157400
C	0.21171000	0.47871300	-0.24795800
C	-0.23761300	-0.85551600	-0.43745200
C	-1.57048400	-1.18887800	-0.45012300
C	-2.57633200	-0.18516400	-0.25648900
N	-3.89442600	-0.48417000	-0.29326000
C	-4.34905100	-1.86839100	-0.43985400
C	-4.88146100	0.56157200	0.03354600
C	-2.13615400	1.14611500	0.01310600
H	2.87347400	2.52318300	0.46275700
H	0.50048100	-1.64817500	-0.54501900
H	-1.84458300	-2.23258400	-0.58994900
H	-5.34667600	-1.84526200	-0.89059700
H	-3.70166400	-2.38034600	-1.16085000
H	-4.68477900	0.89038900	1.06728700
H	-4.67302200	1.42450700	-0.61515900
H	-2.83217300	1.95501600	0.22557700
C	2.64733000	-0.00001400	-0.68461600
O	2.83040500	-1.15847900	0.68776300
C	3.78817800	-2.04065900	0.60672000
O	4.60243200	-2.09406600	-0.31408100
C	3.79678000	-3.02609900	1.74879700
H	2.95489200	-3.72252600	1.61950700

H	4.73577700	-3.59194700	1.75359600
H	3.65377200	-2.50557100	2.70475200
C	-4.37853700	-2.59750600	0.89556000
H	-4.75075800	-3.62051500	0.74722000
H	-3.37105600	-2.65195700	1.33457000
H	-5.04207800	-2.08596000	1.60845000
C	-6.33367700	0.16380400	-0.11828000
H	-6.58231500	-0.10794900	-1.15369100
H	-6.61790600	-0.66228000	0.54834100
H	-6.94420100	1.03580700	0.15324100
O	0.99635700	4.26606300	0.98862800
C	3.95321900	0.63499500	-0.66302600
N	4.98375700	1.17419100	-0.66535700
C	2.44411400	-0.89841300	-1.90181000
H	2.29126100	-1.94871500	-1.61992800
H	1.57452500	-0.55657000	-2.48005700
H	3.33002100	-0.85997500	-2.54804600

PPG 3°-PA (T_1 CIP) optimized geometry [# opt=(calcfc,noeigentest) freq scrf=(smd,solvent=water) def2svp mn15]

EE + Thermal Free Energy Correction: -1105.778473 Ha

0 3

C	2.31955800	1.01522200	0.04831700
C	2.78300700	2.33351300	0.06851500
C	1.91845900	3.49159600	-0.00418000
O	0.58146800	3.25300500	-0.11088100
C	0.05226000	1.99854000	-0.08487700
C	0.87369700	0.83622500	0.01671300
C	0.19230900	-0.40413700	0.11053000
C	-1.17503100	-0.50000000	0.04396200
C	-1.98436100	0.67365100	-0.12187900
N	-3.32649500	0.59809500	-0.21922800
C	-4.01929100	-0.68780600	-0.09804900
C	-4.12321300	1.83956400	-0.28301600
C	-1.31918800	1.93413900	-0.16138700
H	3.84763200	2.56393400	0.11215300
H	0.72612600	-1.33846600	0.25442700
H	-1.61784000	-1.49064100	0.13239700
H	-4.95958700	-0.60969700	-0.65302500
H	-3.42372100	-1.45723100	-0.60113300
H	-3.92083400	2.39888800	0.64456600
H	-3.71834200	2.44045900	-1.10987100
H	-1.85808800	2.87540300	-0.24691300
C	3.28036600	-0.04689400	0.03785600
O	0.18200900	-3.53201000	0.53057600
C	0.67054800	-4.50426000	-0.11420300
O	1.80943900	-4.52608700	-0.64572500
C	-0.22041700	-5.72999400	-0.28246100
H	0.36560800	-6.61805500	-0.55271300

H	-0.79778600	-5.91858400	0.63312600
H	-0.94232800	-5.53262700	-1.09075200
C	-4.27856300	-1.04744000	1.35841500
H	-4.82862000	-1.99720500	1.40081400
H	-3.33259800	-1.16416700	1.90756800
H	-4.88060100	-0.27308100	1.85554600
C	-5.61235200	1.65776700	-0.47299400
H	-5.85224500	1.16759200	-1.42660900
H	-6.07926500	1.09836600	0.34920900
H	-6.06209700	2.65975900	-0.49153900
O	2.29722800	4.64373500	0.00635400
C	4.64604600	0.31851700	0.18766700
N	5.77964700	0.56942000	0.31232800
C	3.04005000	-1.50965500	-0.15193500
H	2.59948200	-1.97008900	0.74791100
H	2.35232400	-1.70171500	-0.98737500
H	3.98626000	-2.02589600	-0.35403400

6. References

(1) KOJU, H. MANUFACTURING METHOD OF 2-[4-(TRIFLUOROMETHYL)PHENYL]ETHYLAMINE AND PYRIMIDINE COMPOUND. JP2018203637A, 2018. https://worldwide.espacenet.com/patent/search/family/064955164/publication/JP2018203637A?q=pn%3DJP2018203637A.

(2) Weinrich, T.; Gränz, M.; Grünewald, C.; Prisner, T. F.; Göbel, M. W. Synthesis of a Cytidine Phosphoramidite with Protected Nitroxide Spin Label for EPR Experiments with RNA. *European J Org Chem* **2017**, *2017* (3), 491–496. https://doi.org/10.1002/ejoc.201601174.

(3) Schulte, A. M.; Alachouzos, G.; Szymański, W.; Feringa, B. L. Strategy for Engineering High Photolysis Efficiency of Photocleavable Protecting Groups through Cation Stabilization. *J Am Chem Soc* **2022**, *144* (27), 12421–12430. https://doi.org/10.1021/jacs.2c04262.

(4) Ahmed, R.; Altieri, A.; DSouza, D. M.; Leigh, D. A.; Mullen, K. M.; Papmeyer, M.; Slawin, A. M. Z.; Wong, J. K. Y.; Woollins, J. D. Phosphorus-Based Functional Groups as Hydrogen Bonding Templates for Rotaxane Formation. *J Am Chem Soc* **2011**, *133* (31), 12304–12310. https://doi.org/10.1021/ja2049786.

(5) Stranius, K.; Börjesson, K. Determining the Photoisomerization Quantum Yield of Photoswitchable Molecules in Solution and in the Solid State. *Sci Rep* **2017**, *7* (1), 1–9. https://doi.org/10.1038/srep41145.

(6) Reddy, K. S.; Yonetani, T.; Tsuneshige, A.; Chance, B.; Kushkuley, B.; Stavrov, S. S.; Vanderkooi, J. M. Infrared Spectroscopy of the Cyanide Complex of Iron(II) Myoglobin and Comparison with Complexes of Microperoxidase and Hemoglobin. *Biochemistry* **1996**, *35* (17), 5562–5570. https://doi.org/10.1021/bi952596m.

(7) Sowole, M. A.; Vuong, S.; Konermann, L. Interactions of Hemoglobin and Myoglobin with Their Ligands CN-, CO, and O2 Monitored by Electrospray Ionization-Mass Spectrometry. *Anal Chem* **2015**, *87* (19), 9538–9545. https://doi.org/10.1021/acs.analchem.5b02506.

(8) Marenich, A. V.; Cramer, C. J.; Truhlar, D. G. Universal Solvation Model Based on Solute Electron Density and on a Continuum Model of the Solvent Defined by the Bulk Dielectric Constant and Atomic Surface Tensions. *Journal of Physical Chemistry B* **2009**, *113* (18), 6378–6396. https://doi.org/10.1021/JP810292N/SUPPL_FILE/JP810292N_SI_003.PDF.

(9) Zheng, J.; Xu, X.; Truhlar, D. G. Minimally Augmented Karlsruhe Basis Sets. *Theor Chem Acc* **2011**, *128* (3), 295–305. https://doi.org/10.1007/S00214-010-0846-Z/TABLES/10.

(10) Yu, H. S.; He, X.; Li, S. L.; Truhlar, D. G. MN15: A Kohn–Sham Global-Hybrid Exchange–Correlation Density Functional with Broad Accuracy for Multi-Reference and Single-Reference Systems and Noncovalent Interactions. *Chem Sci* **2016**, *7* (8), 5032–5051. https://doi.org/10.1039/C6SC00705H.
